# Supplementary material for: Tonsillectomy among children with low baseline acute throat infection consultation rates in UK general practices: a cohort study
Source: BMJ Open. 2015 Feb 3;5(2):e006686. doi: 10.1136/bmjopen-2014-006686 (PMC4322199; doi:10.1136/bmjopen-2014-006686)
Supplement: Author's manuscript [file bmjopen-2014-006686.draft_revisions.pdf]

BMJ Open

Tonsillectomy among children with low baseline acute throat infection consultation rates in UK general practices: a cohort study

Journal:	*BMJ Open*
Manuscript ID:	bmjopen-2014-006686
Article Type:	Research
Date Submitted by the Author:	19-Sep-2014
Complete List of Authors:	Koshy, Elizabeth; Imperial College London, Primary Care and Public Health;<br>Watt, Hilary; Imperial College London, Primary Care and Public Health<br>Curcin, Vasa; Imperial College London, Department of Computing<br>Bottle, Alex; Imperial College, Primary Care and Social Medicine<br>sharland, mike; St Georges Hospital, Paediatric Infectious Diseases Unit<br>Saxena, Sonia; Imperial College London, Primary Care and Social Medicine
**Primary Subject Heading**:	Paediatrics
Secondary Subject Heading:	Ear, nose and throat/otolaryngology, Surgery
Keywords:	Paediatric otolaryngology < PAEDIATRIC SURGERY, PRIMARY CARE, Community child health < PAEDIATRICS

SCHOLARONE™
Manuscripts

For peer review only

Tonsillectomy among children with low baseline acute throat infection consultation rates in UK general practices: a cohort study

Elizabeth Koshy,[1] Hilary Watt,[1] Vasa Curcin,[1] Alex Bottle,[1] Mike Sharland[2] Sonia Saxena[1]

Elizabeth Koshy, MRCGP
Hilary Watt, MSc CStat
Vasa Curcin, PhD
Alex Bottle, PhD
Mike Sharland, FRCPCH
Sonia Saxena, FRCGP

[1] Department of Primary Care and Public Health, Imperial College London
[2] Paediatric Infectious Diseases Unit, St George's Hospital NHS Trust, London

Keywords: tonsillectomy, tonsillitis, child, otorhinolaryngologic diseases, throat

Corresponding author:

Dr. Elizabeth Koshy
Department of Primary Care and Public Health, Imperial College London,
London W6 8RP
Email:e.koshy@imperial.ac.uk

For peer review only - http://bmjopen.bmj.com/site/about/guidelines.xhtml

ABSTRACT

Objective: To investigate the effectiveness of tonsillectomy in reducing acute throat infections (ATI) consultation rates over 6 years' follow-up among children with low baseline ATI consultation rates.

Design: Retrospective cohort study

Setting: UK general practices from the Clinical Practice Research Datalink.

Participants: Children aged 4-15 years with ≤3 ATI consultations during the 3 years prior to 2001 (baseline). 450 children who underwent tonsillectomy and 13,442 other children with an ATI consultation (comparison group) in 2001.

Main outcome measures: Mean differences in ATI consultation rates over the first 3 years' and subsequent 3 years' follow-up compared with 3 years prior to 2001 (baseline); odds of ≥3 ATI consultations at the same time points.

Results: Among children in the tonsillectomy group, the mean ATI consultation rate decreased from 1.31 to 0.66 over the first 3 years' follow-up and further declined to 0.60 over the subsequent 3 years' follow-up period. Compared with children who had no operation, those who underwent tonsillectomy experienced a reduction in mean ATI consultations per child of 2.5 (95% CI 2.3-2.6, P<0.001) over the first 3 years' follow-up, but

For peer review only - http://bmjopen.bmj.com/site/about/guidelines.xhtml

only 1.2 (95% CI 1.0-1.4, P<0.001) over the subsequent 3 years' follow-up compared with baseline, respectively. This equates to a mean reduction of 3.7 ATI consultations over a six year period and approximates to a mean annual reduction of 0.6 ATI consultations per child, per year, over 6 years' follow-up. Children who underwent tonsillectomy were also much less likely to experience ≥3 ATI consultations during the first 3 years' follow-up (OR=0.12, 95% CI: 0.08-0.17) and the subsequent 3 years' follow-up (OR=0.24, 95% CI: 0.14-0.41).

Conclusion: Among children with low baseline ATI rates, there was a statistically significant reduction in ATI consultation rates over 6 years' follow-up. However, the relatively modest clinical benefit needs to be weighed against the potential risks and complications associated with surgery.

For peer review only

Strengths and limitations of study

- This large cohort study is among the first studies to explore the longer-term impact of tonsillectomy among children with low baseline acute throat infection (ATI) consultation rates in a general practice setting in the United Kingdom. We examined ATI consultation history over a 6 year follow-up period using routine, patient-level general practice data from a nationally representative population-based dataset, which limited response bias.

- The main limitations relate to the diagnostic coding of ATIs by clinicians in general practice and that attendance for acute throat infection consultations are influenced by the health-seeking behaviour of parents and management of ATIs by general practitioners.

For peer review only

INTRODUCTION

Tonsillectomy is one of the most common operations performed in children and the majority are performed for frequent acute tonsillitis episodes, for which there is evidence of benefit.[1, 2] [3] [4] However, differentiating between acute tonsillitis, pharyngitis and other causes of acute throat infections (ATI) is challenging in a general practice setting. Children who only have a history of low frequency ATI rates can undergo the operation, despite a lack of evidence for its clinical effectiveness in this group. [1] [5-7] Therefore, in children with low baseline frequency of ATI, clarity is needed about the potential benefits of tonsillectomy over time. Randomised controlled trials (RCT) and previous observational studies have typically not looked beyond two or three years.[4, 7] [8] It is difficult to justify the costs, ethics and logistical problems of mounting new trials over an extended period of time to address this gap in the literature.

The natural history of ATI in children is to spontaneously resolve.[1, 8] This can make it difficult to assess whether tonsillectomy has been effective or whether the ATI frequency would have attenuated over time. Therefore, it is important to compare the prior and subsequent ATI history among children who undergo tonsillectomy with those who do not to evaluate its impact over an extended period of time. Information about the medium and longer-term benefits of tonsillectomy on key outcomes including reduction in subsequent ATI is important for clinicians and to aid parental decision-making. About 98% of children are registered with a general practice in the UK and the vast majority of consultations occur in this setting, which enables real-time ATI consultations to be studied over time.[9, 10]

For peer review only - http://bmjopen.bmj.com/site/about/guidelines.xhtml

Our aim was to investigate whether children with low baseline ATI consultation rates who underwent tonsillectomy experienced a significant reduction in subsequent ATI consultation rates compared with children who did not undergo surgery over six years' follow-up, using longitudinal data from the Clinical Practice Research Datalink (CPRD).

MATERIALS and METHODS

Data source

We used data from the CPRD, which contains individual-level computerised medical records from about 600 general practices and covers data for about 8% of the UK population. [11] The data include demographic details, prescriptions and clinical events, which are entered as diagnostic Read codes by general practitioners during the routine clinical management of patients. CPRD has been extensively used for research.[11]

Study design, ATI definition and study population

We conducted a cohort study of children aged 4-15 years who had <3 ATI consultations at baseline. Children with a record of tonsillectomy or adeno-tonsillectomy between 1st January and 31st December 2001 formed the tonsillectomy group. The comparison group comprised of children with no history of tonsillectomy but a record of a sore throat consultation between these dates (S1). The date for the tonsillectomy record or sore throat consultation in 2001 represented the index date. The study period spanned nine years in total, comprising three years prior to the index date (baseline) and six years subsequent to the index date. The period of follow-up for subsequent consultations was from the index date, which was the date of the first tonsillectomy record or sore throat consultation in 2001, to the date of censoring (the latest CPRD data collection, end of the study period of six

For peer review only - http://bmjopen.bmj.com/site/about/guidelines.xhtml

years post-index date or the patient's transfer out of the practice, whichever came first). We studied ATI consultations prior to and subsequent to the index date.

We developed a list of Read codes (S2) to define prior and subsequent ATI consultations, which encompassed sore throat symptoms and signs, tonsillitis, pharyngitis and also non-specific upper respiratory tract infection (URTI) used by general practitioners to record a throat infection consultation. We searched for non-specific URTI in addition to sore throat consultations, as our previous study showed that the yield can be low by searching exclusively for sore throat consultation record codes. [5]

To enable us to account for prior history of ATI, as a marker of severity, we included children aged 4-15 years if they had ≥3 years' registration prior to the index date (baseline) (S1). We focused our target population on children with low baseline ATIs (with a mild severity spectrum) by excluding those with >3 ATI consultations during the baseline period (1998-2001). Children with a history of snoring, hypertrophied tonsils, sleep apnoea, polysomnography or obstructive sleep apnoea were also excluded from both groups, as this is a different indication for tonsillectomy and our focus was to examine decision-making about tonsillectomy for ATI, not the sleep-disordered breathing (SDB) spectrum. Children aged <4 years were excluded, as pre-school children are more commonly referred for the SDB spectrum than for recurrent ATI.[12] The final study population consisted of 450 children in the tonsillectomy group and 13442 children in the comparison group (S1).

For peer review only - http://bmjopen.bmj.com/site/about/guidelines.xhtml

Outcome measures

Our main outcome was the mean ATI consultation rate per child compared over three time frames across the nine year study period (1998-2007): three years prior to the index date (1998-2001), which is used as a baseline comparator in the main analyses, 1-3 years post-index date (2002-2004) and 4-6 years post-index date (2005-2007) (Table 2). We also calculated the odds of ≥3 versus <3 ATI consultations per child during the same time frames.

Potential risk factors

We examined age, sex, previous ATI consultations and antibiotic history prior to tonsillectomy. Prior ATI consultation frequency was a proxy measure for the severity of ATI infection preceding tonsillectomy. We also examined a number of co-morbid conditions, which are potentially associated with increased risk of URTI and tonsillectomy: asthma, Down's syndrome, cardiac disorders including congenital heart defects, immuno-deficiency, diabetes mellitus, as well as previous inhaled corticosteroid use. [3] [13] We studied practice-level deprivation using 'Index of Multiple Deprivation' scores, based on the location of the general practice. We also investigated *Haemophilus influenza type b* (Hib) immunisation history, as a proxy measure for preventive health-seeking behaviour (HSB).

Statistical analysis

We used Fisher's exact test to investigate the differences in the baseline categorical characteristics of children in both study groups and the unpaired t-test to compare continuous variables. We calculated the mean (with 95% confidence intervals) for ATI

For peer review only - http://bmjopen.bmj.com/site/about/guidelines.xhtml

consultations per child for both groups at: baseline (1998-2001), 1-3 years post-index date (2002-2004) and 4-6 years post-index date (2005-2007), in those with sufficient follow-up. We used the paired t-test to compare the mean differences within each group, followed by the unpaired t-test to compare the mean differences between the two groups. A 2-sided P value of <0.05 was considered to be statistically significant.

Finally, we used logistic regression to investigate the outcomes of: ≥3 versus <3 ATI consultations, in 2002-2004 and 2005-2007 compared with the baseline in 1998-2001, after adjusting for potential confounders. This binary cut-off in ATI consultation frequency was selected to potentially approximate to at least one ATI consultation per year post-index date, respectively, in both time periods. We calculated crude odds ratios (OR) followed by adjusted OR, after controlling for the following potential confounders: age, sex, the co-morbid conditions mentioned above including at least one prior corticosteroid inhaler prescription, prior ATI consultations, prior antibiotics, *Hib* immunisation history and practice-level deprivation, as well as clustering of general practices. We tested for an interaction between age and sex for tonsillectomy *a priori* based on the observations from previous studies. [2, 14] There were no missing data for the socio-demographic variables (age, sex and practice deprivation score). However, the completeness levels of data for the passive smoking and body mass index variables were very poor (<10%), so we excluded these variables and did not adjust for them in the logistic regression analysis. Analysis was performed using Stata/SE version 11.1.[15]

For peer review only - http://bmjopen.bmj.com/site/about/guidelines.xhtml

For peer review only

RESULTS

For the tonsillectomy group, there were 450 children with at least 3 years of prior registration history and ≤3 ATI consultations who underwent tonsillectomy in 2001 (index date). Among these children, 92% (414/450) also had 3 years' follow-up and 80% (358/450) had 6 years' follow-up post-index date, respectively. For the comparison group, there were 13442 children with at least 3 years of prior history and ≤3 ATI consultations. Among these children, 90% (12053/13442) also had 3 years' follow-up and 79% (10593/13442) had 6 years' follow-up post-index date, respectively.

Among the 450 children who underwent tonsillectomy with ≤3 ATI consultations at baseline, the highest percentage of tonsillectomies were performed on children aged 4-7 years (46%, 209/450) and 53% (237/450) were performed on girls. More children received the full vaccination schedule in the tonsillectomy group (55%, 246/450) than those in the comparison group (47%, 6311/13442) and there was evidence of a difference in the total number of Hib vaccinations between the two groups (P<0.001, Table 1).

For peer review only - http://bmjopen.bmj.com/site/about/guidelines.xhtml

Table 1: Characteristics of the study population at baseline (1998-2001) in the tonsillectomy and no tonsillectomy (comparison) groups

	Tonsillectomy ≤3 ATI consultations (N=450)		No Tonsillectomy ≤3 ATI consultations (N=13442)		
					**t-test**
**Mean (SD) Age**	8.6(3.4)		9.3(3.6)		**P<0.001**
**Mean (SD) no. ATI consultations in 3 years prior to index date**	1.3(1.1)		0.4(0.8)		**P<0.001**
**Mean (SD) no. of antibiotics in 3 years prior to index date**	3.7(3.9)		2.1(2.7)		**P<0.001**
	**n**	**%**	**n**	**%**	**χ^2 test**
**Sex**					
**girls**	237	53	7285	54	P=0.52
**boys**	213	47	6157	46	
**Deprivation quintile**					
**1 (least deprived)**	105	23	2848	21	P=0.10
**5 (most deprived)**	103	23	3545	26	
**COMORBIDITIES**					
**asthma**	91	20	2302	17	P=0.10
**cardiac disease and defects**	2	0	11	0	P=0.07
**Down's syndrome**	3	1	15	0	**P=0.02**
**immunodeficiency**	0	0	1	0	P=1.00
**diabetes**	6	1	239	2	P=0.59
**≥ 1 corticosteroid inhaler prescription**	120	27	3259	24	P=0.24
**Hib immunisations**					
**(None) 0**	120	27	4776	36	
**(Partial)1-2**	84	19	2355	18	**P<0.001**
**(Full) 3-4**	246	55	6311	47	

P values shown where statistically significant

ATI – acute throat infection, Hib – H*aemophilus influenza type b*

SD – standard deviation

For peer review only - http://bmjopen.bmj.com/site/about/guidelines.xhtml

Children aged 4-15 years in the tonsillectomy group had a higher mean ATI consultation rate (1.3 over 3 years) than those in the comparison group (0.4 over 3 years) at baseline, 1998-2001 (Table 1). The mean number of baseline antibiotic prescriptions was also higher in the tonsillectomy group (3.7 and 2.1 prescriptions over the 3 years) in the tonsillectomy and comparison groups, respectively, Table 1).

In the tonsillectomy group, the mean ATI consultation rate among children aged 4-15 years decreased from 1.31 to 0.66 between 2002-2004 and 1998-2001, and was 0.60 in 2005-2007 (Table 2). This represented a difference in mean ATI consultation rate of -0.65 (95% CI -0.78 to -0.51) between 1998-2001 and 2002-2004, and then of -0.72 (95% CI -0.88 to -0.56) in 2005-2007 compared with the same baseline (1998-2001).

By contrast, in the comparison group, the mean ATI consultation rate increased from 0.44 to 2.25 between 1998-2001 and 2002-2004, and was 0.93 in 2005-2007 (Table 2). This represented a difference in mean ATI consultation rate of +1.81 (95% CI 1.78 to 1.84) between 1998-2001 and 2002-2004, and then of +0.49 (95% CI 0.46 to 0.52) in 2005-2007 compared with 1998-2001.

Among children aged 4-15 years, comparing those who underwent tonsillectomy with those who did not, the unpaired t-test showed a net reduction in mean ATI consultations per child of 2.46 (95% CI 2.29 to 2.63, P<0.001) over 1-3 years' follow-up and of 1.21 (95% CI 1.04 to 1.38, P<0.001) over 4-6 years' follow-up, compared with baseline. This represents a net

For peer review only - http://bmjopen.bmj.com/site/about/guidelines.xhtml

reduction of 3.67 ATI consultations over 6 years' follow-up or approximates to an annual reduction in mean ATI consultations of 0.61 per child, per year, over 6 years' follow-up.

For peer review only

For peer review only - http://bmjopen.bmj.com/site/about/guidelines.xhtml

Table 2 : Mean differences in acute throat infection (ATI) consultations per child prior to and subsequent to the index date in 2001

	mean number of ATI consultations and mean differences (with 95% CIs) comparing 3 year time intervals (1998-2001, 2002-2004 and 2005-2007)							
	n	1998-2001 3 years prior to index	2002- 2004 1-3 years post index	mean difference between 1998-2001 and 2002-2004 &	n	1998-2001 3 years prior to index	2005-2007 4-6 years post index	mean difference between 1998-2001 and 2005-2007&
	3 years prior to index date and 1-3 years follow up				3 years prior to index date and 4-6 years follow up			
**ALL CHILDREN**								
No Tonsillectomy	12053	0.44	2.25	**+1.81 (1.78 to 1.84)** ***	10593	0.44	0.93	**+0.49 (0.46** to **0.52)***
Tonsillectomy	414	1.31	0.66	**-0.65 (-0.78 to -0.51)***	358	1.32	0.6	**-0.72 (-0.88 to -0.56)***
**GIRLS 4-7 years**								
No Tonsillectomy	2375	0.7	2.43	**+1.74 (1.66 to 1.81)***	2092	0.7	1.04	**+0.35 (0.27 to 0.42)***
Tonsillectomy	78	1.32	0.76	**-0.56 (-0.94 to -0.19)**	71	1.34	0.65	**-0.69 (-1.08 to -0.31)***
**BOYS 4-7 years**								
No Tonsillectomy	2242	0.7	2.29	**+1.59 (1.51 to 1.67)***	1989	0.69	0.76	+0.06 (0.00 to 0.13)
Tonsillectomy	110	1.32	0.83	**-0.49 (-0.81 to -0.18)**	92	1.32	0.55	**-0.76 (-1.02 to -0.50)***
**GIRLS 8-11 years**								
No Tonsillectomy	1944	0.35	2.23	**+1.87 (1.80 to 1.95)***	1744	0.35	1.08	**+0.73 (0.65 to 0.81)***
Tonsillectomy	71	1.43	0.73	**-0.70 (-0.99 to -0.42)***	65	1.43	0.92	**-0.51 (-1.00 to -0.01)***
**BOYS 8-11 years**								
No Tonsillectomy	1526	0.31	2.04	**+1.73 (1.65 to 1.80)***	1395	0.31	0.71	**+0.40 (0.33 to 0.47)***
Tonsillectomy	59	1.17	0.34	**-0.83 (-1.16 to -0.51)***	49	1.22	0.39	**-0.84 (-1.23 to -0.45)***
**GIRLS 12-15 years**								
No Tonsillectomy	2206	0.23	2.45	**+2.22 (2.14 to 2.30)***	1866	0.23	1.2	**+0.97 (0.89 to 1.04)***
Tonsillectomy	66	1.29	0.62	**-0.67 (-0.95 to -0.38)***	55	1.22	0.62	**-0.60 (-0.96 to -0.24)**
**BOYS 12-15 years**								
No Tonsillectomy	1760	0.2	1.91	**+1.70 (1.64 to 1.77)***	1507	0.21	0.68	**+0.47 (0.41 to 0.53)***
Tonsillectomy	30	1.27	0.37	**-0.90 (-1.28 to -0.52)***	26	1.42	0.23	**-1.19 (-1.69 to -0.69)***

& paired t-test
* P <0.05
** P<0.01
*** P<0.001

The crude odds ratio (OR) for the effect of tonsillectomy on the outcome of ≥3 ATI consultations over 1-3 years' follow-up was 0.18 (95% CI: 0.13-0.27) and the adjusted OR was 0.12 (95% CI: 0.08-0.17). The crude OR for ≥3 ATI consultations over 4-6 years' follow-up was 0.36 (95% CI: 0.22-0.59), whilst the adjusted OR was 0.24 (95% CI: 0.14-0.41). The crude odds ratios were adjusted for age, sex, the co-morbid conditions mentioned earlier, including at least one prior corticosteroid inhaler prescription, prior ATI consultations, prior antibiotics, *Hib* immunisation history, practice-level deprivation and clustering of general practices. Prior ATI consultations, followed by prior antibiotics, were the strongest confounders of the association between tonsillectomy and the outcome of ATI consultations.

DISCUSSION

Synopsis of key findings

Children aged 4-15 years with a history of low ATI consultation frequency at baseline (≤3 ATI consultations during three years) who underwent tonsillectomy experienced a net reduction in the mean ATI consultation rate of 3.67 over six years' follow up, representing 2.46 (95% CI 2.29 to 2.63, P<0.001) over the first three years and 1.21 (95% CI 1.04 to 1.38, P<0.001) per child over the subsequent three years' follow-up, compared with those who did not undergo surgery. This approximates to an annual reduction in the mean ATI consultation rate of 0.61 per child, per year, over six years' follow-up. Tonsillectomy was also associated with much lower odds of ≥3 ATI consultations throughout the six year follow-up period.

For peer review only

Strengths and limitations of the study

This is among the first studies to explore the longer-term impact of tonsillectomy in a general practice setting in the UK, covering nine years. We used routine individual-level general practice data from a nationally representative population-based dataset, which limited response bias. The large number of comparisons from a wide population base increased the power of the study.

However, there are also a number of limitations to our study. Firstly, our measure of ATI frequency relied on visits to the general practitioner rather than self-reporting. Although this is an objective measure, we recognise it relies on health-seeking behaviour and could, for example, underestimate the frequency of ATI episodes since not every child attends with every ATI episode. Secondly, although we endeavoured to exclude all children who may have undergone tonsillectomy for the SDB spectrum, we are unlikely to have captured everyone who had surgery for this indication. Thirdly, the losses to follow-up were similar in the tonsillectomy and comparison groups over the three and six year follow-up periods and were both within 20% at six years' follow-up. Losses to follow-up could have created attrition bias, but this is difficult to minimise and this also affects experimental studies. Fourthly, there were higher baseline ATI consultation and antibiotic means in the tonsillectomy group. However, we adjusted for prior ATI consultations and antibiotics which made the two groups more comparable. Finally, the large size of the study population with demographic and co-morbidity variables, which were available from the CPRD dataset, allowed us to adjust for a number of potential confounding factors. However, as with most retrospective cohort studies, we were unable to study all known and unknown potential

For peer review only - http://bmjopen.bmj.com/site/about/guidelines.xhtml

confounding variables and thus there could be residual confounding, which we could not control for.

Comparisons with other studies

A Dutch RCT among children aged 2-8 years who did not fulfil the Paradise criteria [4] for severe recurrent throat infection frequency, which found the effects of tonsillectomy were more pronounced in children who had 3-6 throat infections in the year prior to entering the trial than in those with ≤2 throat infections.[8] Among children in this latter group, the authors reported fewer throat infections (-0.21, 95% CI -0.06 to -0.36) and fewer sore throats (-0.60, 95% CI -0.30 to -0.90) per person year in the tonsillectomy group but concluded that these differences were not large enough to be clinically significant, although the median follow-up time was only 22 months. [8] Although ours was a cohort study, we similarly found that although tonsillectomy led to a statistically significant reduction in ATI consultation rates among children with low baseline ATI, this was not necessarily clinically significant.

We found the natural history among children of all ages who did not have tonsillectomy was for a gradual increase in the number of ATI consultations. This is contrary to previous studies, which reported that the natural history of sore throats is to attenuate over time.[16] [17, 18] However, our study was based in a community setting and focused on a population of children with low baseline ATI who may have emerging natural history whereas other studies have selected children with higher baseline ATI from the outset. If we had followed children up for longer we may have observed a decline over time.

For peer review only - http://bmjopen.bmj.com/site/about/guidelines.xhtml

Clinical implications of the study

Overall, children aged 4-15 years, who underwent tonsillectomy compared with those who did not, experienced an 88% and 76% reduced risk of having ≥3 ATI consultations over 1-3 years and 4-6 years' follow-up, respectively. However, the observed decline in ATI consultations might also, in part, be attributed to adeno-tonsillectomy operations reducing rhino-sinusitis or otitis media infections, which were coded as URTIs.[19, 20] [21]

Higher Hib vaccinations were also associated with tonsillectomy, which suggests those with higher levels of preventive health-seeking behaviour (HSB) may have been more likely to undergo tonsillectomy. There was no evidence of an association between tonsillectomy and deprivation level, so it is unclear whether those with higher preventive HSB represented the least or most deprived families. However, we only had practice-level deprivation data and not individual-level data, which could account for the absence of a deprivation gradient.

Children in our study who underwent tonsillectomy in 2001 had ≤3 ATI consultations documented in general practice during the three years prior to their operation. This is markedly less than the minimum threshold recommended by national guidelines, reviews and the Paradise criteria for frequency of episodes prior to tonsillectomy. [1] [4] [18] [22] [23] These findings may reflect lower thresholds being followed or perhaps represent sub-optimal documentation of sore throat consultations prior to tonsillectomy in primary care settings. Although we assumed that parents would have consulted if the ATI was severe, the low consultation rate could reflect HSB, whereby some parents may not present with ATI

For peer review only - http://bmjopen.bmj.com/site/about/guidelines.xhtml

episodes if, for example, antibiotics were not issued at prior consultations. However, this depends on the individual GP's attitudes and threshold for prescribing antibiotics for ATIs. Other possible explanations are that we did not have access to the 'free text' within individual medical consultation records or direct Accident and Emergency department attendances or admissions, which have been increasing in recent years.[24] Therefore, we recommend future studies should examine individual consultations in greater detail, as well as attendances in community and hospital emergency settings.

Tonsillectomy is a major operation so it is important that surgery confers significant individual and societal benefits, including fewer days off work by parents affected by sick children. Previous research has suggested surgery is beneficial in the short-term for those with more serious ATI morbidity at baseline. However, its benefit in the longer-term is still not clear and this still needs to be investigated.

Conclusion

Our study found a statistically significant reduction in ATI consultation rates among children with low baseline ATI consultation rates who underwent tonsillectomy compared with those who did not. However, for this subgroup of children, the relatively modest reduction in ATI consultation rates in clinical terms may be outweighed by the potential risks related to the operation. The long-term clinical effectiveness of tonsillectomy for children, across the ATI frequency spectrum, still needs to be evaluated. This needs to undertaken in conjunction with updated cost-effectiveness models, stratified by age, sex and baseline ATI rates, so that

For peer review only - http://bmjopen.bmj.com/site/about/guidelines.xhtml

clinicians can risk profile children to determine which subgroups of children are most likely to benefit from surgery.

For peer review only

For peer review only - http://bmjopen.bmj.com/site/about/guidelines.xhtml

FUNDING: EK is funded by a National Institute for Health Research Doctoral Research Fellowship (2009-02-78). SS is funded by a a National Institute for Health Research Career Development Fellowship (NIHR CDF-2011-04-048). This article presents independent research commissioned by the National Institute for Health Research (NIHR) under the Collaborations for Leadership in The views expressed in this publication are those of the author(s) and not necessarily those of the NHS, the NIHR or the Department of Health.

ETHICAL APPROVAL : The study was approved by the MHRA Independent Scientific Advisory Committee on Database Research.

ACKNOWLEDGEMENTS: Data was obtained from the Clinical Practice Research Datalink, CPRD (formerly known as the General Practice Research Database). The CPRD is jointly funded by the NHS National Institute for Health Research (NIHR) and the Medicines and Healthcare products Regulatory Agency (MHRA). EK is funded by a National Institute for Health Research Doctoral Research Fellowship. SS is funded by a National Institute for Health Research Senior Researcher fellowship. HW is funded by NIHR Research Design Service.

COMPETING INTERESTS: none declared

DATA SHARING STATEMENT: no additional data are available

CONTRIBUTOR STATEMENT: The study was conceived by EK, SS and MS. The study was designed by EK, SS, HW and AB. VC extracted the data. EK analysed the data. HW provided statistical advice. EK wrote the first draft of the manuscript. All authors contributed to the interpretation of the findings and revised the paper critically for intellectual content. All authors have seen and approved this version of the manuscript. EK and SS are the guarantors of this study.

For peer review only - http://bmjopen.bmj.com/site/about/guidelines.xhtml

For peer review only

REFERENCES

1. SIGN. Scottish Intercollegiate Guidelines Network 117. Management of sore throat and indications for tonsillectomy. April 2010.

2. Lock C, Wilson J, Steen N, et al. North of England and Scotland Study of Tonsillectomy and Adeno-tonsillectomy in Children(NESSTAC): a pragmatic randomised controlled trial with a parallel non-randomised preference study. Health Technol Assess. 2010 Mar;14(13):1-164, iii-iv.

3. Orvidas LJ, St Sauver JL, Weaver AL. Efficacy of tonsillectomy in treatment of recurrent group A beta-hemolytic streptococcal pharyngitis. Laryngoscope. 2006 Nov;116(11):1946-50.

4. Paradise JL, Bluestone CD, Bachman RZ, et al. Efficacy of tonsillectomy for recurrent throat infection in severely affected children. Results of parallel randomized and nonrandomized clinical trials. N Engl J Med. 1984 Mar 15;310(11):674-83.

5. Koshy E, Curcin V, Bottle A, et al. Sore throat consultations in general practice prior to tonsillectomy among eight hundred and sixty-three children in England: is this in accordance with the SIGN guidelines? Clin Otolaryngol. 2013 Jun;38(3):266-70.

6. Toh A, Mullin A, Grainger J, et al. Indications for tonsillectomy: are we documenting them? Annals of the Royal College of Surgeons of England. 2009 Nov;91(8):697-9.

For peer review only

For peer review only - http://bmjopen.bmj.com/site/about/guidelines.xhtml

7. Paradise JL, Bluestone CD, Colborn DK, et al. Tonsillectomy and adenotonsillectomy for recurrent throat infection in moderately affected children. Pediatrics. 2002 Jul;110(1 Pt 1):7-15.

8. van Staaij BK, van den Akker EH, Rovers MM, et al. Effectiveness of adenotonsillectomy in children with mild symptoms of throat infections or adenotonsillar hypertrophy: open, randomised controlled trial. BMJ. 2004 Sep 18;329(7467):651.

9. Hippisley-Cox J, Vinogradova Y. Trends in Consultation Rates in General Practice 1995/1996 to 2008/2009: Analysis of the QResearch® database. http://www.ic.nhs.uk/webfiles/publications/gp/Trends_in_Consultation_Rates_in_General_Practice_1995_96_to_2008_09.pdf. 2009.

10. Saxena S, Francis N, Sharland M. Primary care of children: the unique role of GPs. Br J Gen Pract. 2012 Jul;62(600):340-1.

11. CPRD. Clinical Practice Research Datalink. http://www.cprd.com/.

12. Parker NP, Walner DL. Trends in the indications for pediatric tonsillectomy or adenotonsillectomy. Int J Pediatr Otorhinolaryngol. 2011 Feb;75(2):282-5.

13. Ram G, Chinen J. Infections and immunodeficiency in Down syndrome. Clinical and experimental immunology. 2011 Apr;164(1):9-16.

For peer review only

For peer review only - http://bmjopen.bmj.com/site/about/guidelines.xhtml

14. Vestergaard H, Wohlfahrt J, Westergaard T, et al. Incidence of tonsillectomy in Denmark, 1980 to 2001. Pediatr Infect Dis J. 2007 Dec;26(12):1117-21.

15. Stata/SE version 11.1 http://www.stata.com/stata11/point1.html.

16. Buskens E, van Staaij B, van den Akker J, et al. Adenotonsillectomy or watchful waiting in patients with mild to moderate symptoms of throat infections or adenotonsillar hypertrophy: a randomized comparison of costs and effects. Arch Otolaryngol Head Neck Surg. 2007 Nov;133(11):1083-8.

17. van Staaji BK, van den Akker EH, Rovers MM, et al. Effectiveness of adenotonsillectomy in children with mild symptoms of throat infections or adenotonsillar hypertrophy: open, randomised controlled trial. Clin Otolaryngol. 2005 Feb;30(1):60-3.

18. Burton MJ, Glasziou PP. Tonsillectomy or adeno-tonsillectomy versus non-surgical treatment for chronic/recurrent acute tonsillitis. Cochrane Database Syst Rev. 2009(1):CD001802.

19. van den Aardweg MT, Schilder AG, Herkert E, et al. Adenoidectomy for otitis media in children. Cochrane Database Syst Rev. 2010(1):CD007810.

20. Paradise JL, Bluestone CD, Colborn DK, et al. Adenoidectomy and adenotonsillectomy for recurrent acute otitis media: parallel randomized clinical trials in children not previously treated with tympanostomy tubes. JAMA. 1999 Sep 8;282(10):945-53.

For peer review only - http://bmjopen.bmj.com/site/about/guidelines.xhtml

21. Darrow DH, Siemens C. Indications for tonsillectomy and adenoidectomy. Laryngoscope. 2002 Aug;112(8 Pt 2 Suppl 100):6-10.

22. Baugh RF, Archer SM, Mitchell RB, et al. Clinical practice guideline: tonsillectomy in children. Otolaryngol Head Neck Surg. 2011 Jan;144(1 Suppl):S1-30.

23. SIGN. Scottish Intercollegiate Guidelines Network 34: Management of sore throat and indications for tonsillectomy. 1999. Available on specific request, as it is no longer accessible on the SIGN website.

.

24. Koshy E, Murray J, Bottle A, et al. Significantly increasing hospital admissions for acute throat infections among children in England: is this related to tonsillectomy rates? Arch Dis Child. 2012 Dec;97(12):1064-8.

For peer review only

S1: Flow diagram of cohort construction

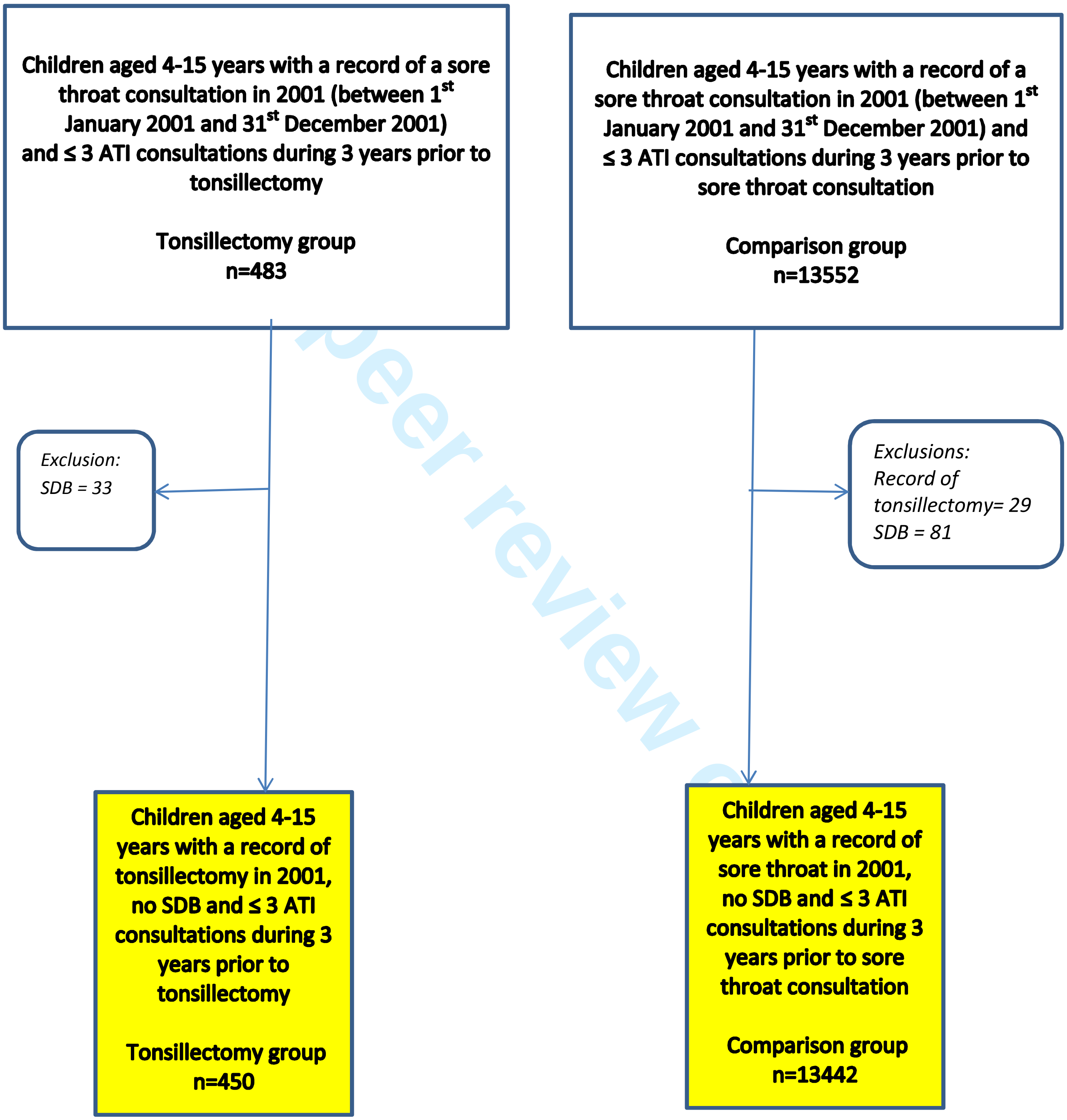

ATI- acute throat infection; SDB – sleep- disordered breathing

For peer review only - http://bmjopen.bmj.com/site/about/guidelines.xhtml

S2 – Read codes

Sore throat

Acute tonsillitis	H03
Acute erythematous tonsillitis	H030.
Acute follicular tonsillitis	H031.
Acute ulcerative tonsillitis	H032.
Acute catarrhal tonsillitis	H033.
Acute gangrenous tonsillitis	H034.
Acute bacterial tonsillitis	H035.
Acute viral tonsillitis	H036.
Acute tonsillitis NOS	H03z
streptococcal tonsillitis	A3403
Acute staphylococcal tonsillitis	H0351
Acute pneumococcal tonsillitis	H0350
O/E - exudate on tonsils	2DB7.
Acute pharyngitis	H02.
Acute gangrenous pharyngitis	H020.
Acute ulcerative pharyngitis	H022.
Acute bacterial pharyngitis	H023.
Acute viral pharyngitis	H024.
Acute staphylococcal pharyngitis	H0231
Acute pharyngitis NOS	H02z
Acute bacterial pharyngitis NOS	H023z
Streptococcal pharyngitis	A3402
Acute pneumococcal pharyngitis	H0230
Acute phlegmonous pharyngitis	H021.
Acute nasopharyngitis	H00

For peer review only - http://bmjopen.bmj.com/site/about/guidelines.xhtml

Streptococcal sore throat	A340.
Has a sore throat	1C92.
Persistent sore throat	1C93.
Sore throat symptom NOS	1C9Z.
Streptococcal sore throat NOS	A340z
throat pain	1CB3.
[D]Throat pain	R041.
Throat soreness	1C9..
Has a sore throat	1C92.

Upper respiratory tract infections

H1z..00	Upper respiratory tract disease NOS
H051.00	Acute upper respiratory tract infection
H05z.11	Upper respiratory tract infection NOS
H05..00	Other acute upper respiratory infections
H05z.12	Viral upper respiratory tract infection NOS
H05z.00	Upper respiratory infection NOS

For peer review only - http://bmjopen.bmj.com/site/about/guidelines.xhtml

STROBE 2007 (v4) checklist of items to be included in reports of observational studies in epidemiology*
Checklist for cohort, case-control, and cross-sectional studies (combined)

Section/Topic	Item #	Recommendation	Reported on page #
**Title and abstract**	1	(*a*) Indicate the study's design with a commonly used term in the title or the abstract	1
		(*b*) Provide in the abstract an informative and balanced summary of what was done and what was found	2-3
**Introduction**			
Background/rationale	2	Explain the scientific background and rationale for the investigation being reported	5
Objectives	3	State specific objectives, including any pre-specified hypotheses	5
**Methods**			
Study design	4	Present key elements of study design early in the paper	6-7
Setting	5	Describe the setting, locations, and relevant dates, including periods of recruitment, exposure, follow-up, and data collection	6-8
Participants	6	(*a*) *Cohort study*—Give the eligibility criteria, and the sources and methods of selection of participants. Describe methods of follow-up<br>*Case-control study*—Give the eligibility criteria, and the sources and methods of case ascertainment and control selection. Give the rationale for the choice of cases and controls<br>*Cross-sectional study*—Give the eligibility criteria, and the sources and methods of selection of participants	6-7
		(*b*) *Cohort study*—For matched studies, give matching criteria and number of exposed and unexposed<br>*Case-control study*—For matched studies, give matching criteria and the number of controls per case	n/a
Variables	7	Clearly define all outcomes, exposures, predictors, potential confounders, and effect modifiers. Give diagnostic criteria, if applicable	7-8
Data sources/ measurement	8*	For each variable of interest, give sources of data and details of methods of assessment (measurement). Describe comparability of assessment methods if there is more than one group	*6-7*
Bias	9	Describe any efforts to address potential sources of bias	7,9
Study size	10	Explain how the study size was arrived at	6
Quantitative variables	11	Explain how quantitative variables were handled in the analyses. If applicable, describe which groupings were chosen and why	8-9
Statistical methods	12	(*a*) Describe all statistical methods, including those used to control for confounding	8-9
		(*b*) Describe any methods used to examine subgroups and interactions	8-9
		(*c*) Explain how missing data were addressed	9
		(*d*) *Cohort study*—If applicable, explain how loss to follow-up was addressed<br>*Case-control study*—If applicable, explain how matching of cases and controls was addressed	16

For peer review only

		Cross-sectional study—If applicable, describe analytical methods taking account of sampling strategy	
		(*e*) Describe any sensitivity analyses	
**Results**			
Participants	13*	(a) Report numbers of individuals at each stage of study—eg numbers potentially eligible, examined for eligibility, confirmed eligible, included in the study, completing follow-up, and analysed	10, S2
		(b) Give reasons for non-participation at each stage	10
		(c) Consider use of a flow diagram	S2
Descriptive data	14*	(a) Give characteristics of study participants (eg demographic, clinical, social) and information on exposures and potential confounders	11
		(b) Indicate number of participants with missing data for each variable of interest	9
		(c) *Cohort study*—Summarise follow-up time (eg, average and total amount)	9-10
Outcome data	15*	*Cohort study*—Report numbers of outcome events or summary measures over time	12-14
		Case-control study—Report numbers in each exposure category, or summary measures of exposure	
		Cross-sectional study—Report numbers of outcome events or summary measures	
Main results	16	(*a*) Give unadjusted estimates and, if applicable, confounder-adjusted estimates and their precision (eg, 95% confidence interval). Make clear which confounders were adjusted for and why they were included	15 and 9
		(*b*) Report category boundaries when continuous variables were categorized	11
		(*c*) If relevant, consider translating estimates of relative risk into absolute risk for a meaningful time period	12
Other analyses	17	Report other analyses done—eg analyses of subgroups and interactions, and sensitivity analyses	
**Discussion**			
Key results	18	Summarise key results with reference to study objectives	15
Limitations	19	Discuss limitations of the study, taking into account sources of potential bias or imprecision. Discuss both direction and magnitude of any potential bias	16-17
Interpretation	20	Give a cautious overall interpretation of results considering objectives, limitations, multiplicity of analyses, results from similar studies, and other relevant evidence	16-19
Generalisability	21	Discuss the generalisability (external validity) of the study results	17-19
**Other information**			
Funding	22	Give the source of funding and the role of the funders for the present study and, if applicable, for the original study on which the present article is based	21

*Give information separately for cases and controls in case-control studies and, if applicable, for exposed and unexposed groups in cohort and cross-sectional studies.

Note: An Explanation and Elaboration article discusses each checklist item and gives methodological background and published examples of transparent reporting. The STROBE checklist is best used in conjunction with this article (freely available on the Web sites of PLoS Medicine at http://www.plosmedicine.org/, Annals of Internal Medicine at http://www.annals.org/, and Epidemiology at http://www.epidem.com/). Information on the STROBE Initiative is available at www.strobe-statement.org.

For peer review only

BMJ Open

Tonsillectomy among children with low baseline acute throat infection consultation rates in UK general practices: a cohort study

Journal:	*BMJ Open*
Manuscript ID:	bmjopen-2014-006686.R1
Article Type:	Research
Date Submitted by the Author:	28-Oct-2014
Complete List of Authors:	Koshy, Elizabeth; Imperial College London, Primary Care and Public Health;<br>Watt, Hilary; Imperial College London, Primary Care and Public Health<br>Curcin, Vasa; Kings College London, Primary Care and Public Health Sciences<br>Bottle, Alex; Imperial College, Primary Care and Social Medicine<br>sharland, mike; St George's University London, Paediatric Infectious Diseases Research Group<br>Saxena, Sonia; Imperial College London, Primary Care and Social Medicine
**Primary Subject Heading**:	Paediatrics
Secondary Subject Heading:	Ear, nose and throat/otolaryngology, Surgery
Keywords:	Paediatric otolaryngology < PAEDIATRIC SURGERY, PRIMARY CARE, Community child health < PAEDIATRICS

SCHOLARONE™
Manuscripts

For peer review only

Tonsillectomy among children with low baseline acute throat infection consultation rates in UK general practices: a cohort study

Elizabeth Koshy,[1] Hilary Watt,[1] Vasa Curcin,[2] Alex Bottle,[1] Mike Sharland[3] Sonia Saxena[1]

Elizabeth Koshy, MRCGP
Hilary Watt, MSc CStat
Vasa Curcin, PhD
Alex Bottle, PhD
Mike Sharland, FRCPCH
Sonia Saxena, FRCGP

[1] Department of Primary Care and Public Health, Imperial College London
[2] Department of Primary Care and Public Health Sciences, Kings College London
[3] Paediatric Infectious Diseases Research Group, St George's University London, London

Keywords: tonsillectomy, tonsillitis, child, otorhinolaryngologic diseases, throat

Corresponding author:

Dr. Elizabeth Koshy
Department of Primary Care and Public Health, Imperial College London,
London W6 8RP
Email:e.koshy@imperial.ac.uk

For peer review only - http://bmjopen.bmj.com/site/about/guidelines.xhtml

ABSTRACT

Objective: To investigate the effectiveness of tonsillectomy in reducing acute throat infection (ATI) consultation rates over 6 years' follow-up among children with low baseline ATI consultation rates.

Design: Retrospective cohort study

Setting: UK general practices from the Clinical Practice Research Datalink.

Participants: Children aged 4-15 years with ≤3 ATI consultations during the 3 years prior to 2001 (baseline). 450 children who underwent tonsillectomy (tonsillectomy group) and 13,442 other children with an ATI consultation (comparison group) in 2001.

Main outcome measures: Mean differences in ATI consultation rates over the first 3 years' and subsequent 3 years' follow-up compared with 3 years prior to 2001 (baseline); odds of ≥3 ATI consultations at the same time points.

Results: Among children in the tonsillectomy group, the 3-year mean ATI consultation rate decreased from 1.31 to 0.66 over the first 3 years' follow-up and further declined to 0.60 over the subsequent 3 years' follow-up period. Compared with children who had no operation, those who underwent tonsillectomy experienced a reduction in 3-year mean ATI consultations per child of 2.5 (95% CI 2.3-2.6, P<0.001) over the first 3 years' follow-up, but

For peer review only

only 1.2 (95% CI 1.0-1.4, P<0.001) over the subsequent 3 years' follow-up compared with baseline, respectively. This equates to a mean reduction of 3.7 ATI consultations over a six year period and approximates to a mean annual reduction of 0.6 ATI consultations per child, per year, over 6 years' follow-up. Children who underwent tonsillectomy were also much less likely to experience ≥3 ATI consultations during the first 3 years' follow-up (adjusted OR=0.12, 95% CI:0.08-0.17) and the subsequent 3 years' follow-up (adjusted OR=0.24, 95% CI:0.14-0.41).

Conclusion: Among children with low baseline ATI rates, there was a statistically significant reduction in ATI consultation rates over 6 years' follow-up. However, the relatively modest clinical benefit needs to be weighed against the potential risks and complications associated with surgery.

For peer review only

For peer review only - http://bmjopen.bmj.com/site/about/guidelines.xhtml

Strengths and limitations of study

- This large cohort study is among the first to explore the longer-term impact of tonsillectomy among children with low baseline acute throat infection (ATI) consultation rates in a general practice setting in the United Kingdom. We examined ATI consultation history over a 6 year follow-up period using routine, patient-level general practice data from a nationally representative population-based dataset, which limited response bias.

- The main limitations relate to the diagnostic coding of ATIs by clinicians in general practice and that attendance for acute throat infection consultations is influenced by the health-seeking behaviour of parents and the management of ATIs by general practitioners.

For peer review only - http://bmjopen.bmj.com/site/about/guidelines.xhtml

INTRODUCTION

Tonsillectomy is one of the most common operations performed in children and the majority are performed for frequent acute tonsillitis episodes, for which there is evidence of benefit.[1, 2] [3] [4] However, differentiating between acute tonsillitis, pharyngitis and other causes of acute throat infections (ATI) is challenging in a general practice setting. Children who only have a record of low frequency ATI rates can undergo the operation, despite a lack of evidence for its clinical effectiveness in this group. [1] [5-8] Therefore, in children with low baseline frequency of ATI, clarity is needed about the potential benefits of tonsillectomy over time. Randomised controlled trials (RCT) and previous observational studies have typically not looked beyond two or three years.[3, 4, 7] [8, 9] It is difficult to justify the costs, ethics and logistical problems of mounting new trials over an extended period of time to address this gap in the literature.

The natural history of ATI in children is to spontaneously resolve.[1, 8] This can make it difficult to assess whether tonsillectomy has been effective or whether the ATI frequency would have attenuated over time. Therefore, it is important to compare the prior and subsequent ATI history among children who undergo tonsillectomy with those who do not to evaluate its impact over an extended period of time. Information about the medium and longer-term benefits of tonsillectomy on key outcomes including reduction in subsequent ATI is important for clinicians and to aid parental decision-making. About 98% of children are registered with a general practice in the UK and the vast majority of consultations occur in this setting, which enables real-time ATI consultations to be studied over time.[10, 11]

For peer review only - http://bmjopen.bmj.com/site/about/guidelines.xhtml

Our aim was to investigate whether children with low baseline ATI consultation rates who underwent tonsillectomy experienced a significant reduction in subsequent ATI consultation rates compared with children who did not undergo surgery over six years' follow-up, using longitudinal data from the Clinical Practice Research Datalink (CPRD) database.

MATERIALS and METHODS

Data source

We used data from the CPRD, which contains individual-level computerised medical records from about 600 general practices and covers data for about 8% of the UK population. [12] The data include demographic details, prescriptions and clinical events, which are entered as diagnostic Read codes by general practitioners during the routine clinical management of patients. CPRD has been extensively used for research.[12]

Study design, ATI definition and study population

We conducted a cohort study of children aged 4-15 years who had ≤3 ATI consultations at baseline. Children with a record of tonsillectomy or adeno-tonsillectomy between 1st January and 31st December 2001 formed the tonsillectomy group. The comparison group comprised of children with no history of tonsillectomy but a record of a sore throat consultation between these dates (supplementary figure S1). The date for the tonsillectomy record or sore throat consultation in 2001 represented the index date. The study period spanned nine years in total, comprising three years prior to the index date (baseline) and six years subsequent to the index date. The period of follow-up for subsequent consultations was from the index date, which was the date of the first tonsillectomy record or sore throat consultation in 2001, to the date of censoring (the latest CPRD data collection, end of the

For peer review only - http://bmjopen.bmj.com/site/about/guidelines.xhtml

study period of six years post-index date or the patient's transfer out of the practice, whichever came first). We studied ATI consultations prior to and subsequent to the index date.

We developed a list of Read codes (supplementary table S2) to define prior and subsequent ATI consultations, which encompassed sore throat symptoms and signs, tonsillitis, pharyngitis and also non-specific upper respiratory tract infection (URTI) codes used by general practitioners to record a throat infection consultation. We searched for non-specific URTI in addition to sore throat consultations, as our previous study showed that the yield can be low by searching exclusively for sore throat consultation record codes. [5]

To enable us to account for prior history of ATI, as a marker of severity, we included children aged 4-15 years if they had ≥3 years' registration prior to the index date (baseline) (supplementary figure S1). We focused our target population on children with low baseline ATIs (with a mild severity spectrum) by excluding those with >3 ATI consultations during the baseline period (1998-2001). Children with a history of snoring, hypertrophied tonsils, sleep apnoea, polysomnography or obstructive sleep apnoea were also excluded from both groups, as this is a different indication for tonsillectomy and our focus was to examine decision-making about tonsillectomy for ATI, not the sleep-disordered breathing (SDB) spectrum. Children aged <4 years were excluded, as pre-school children are more commonly referred for the SDB spectrum than for recurrent ATI.[13] The final study population consisted of 450 children in the tonsillectomy group and 13442 children in the comparison group (supplementary figure S1).

For peer review only - http://bmjopen.bmj.com/site/about/guidelines.xhtml

Outcome measures

Our main outcome was the mean ATI consultation rate per child compared over three time frames across the nine year study period (1998-2007): three years prior to the index date (1998-2001), which is used as a baseline comparator in the main analyses, 1-3 years' follow-up post-index date (2002-2004) and 4-6 years' follow-up post-index date (2005-2007) (Table 2). We also calculated the odds of ≥3 versus <3 ATI consultations per child during the same follow-up time frames.

Potential risk factors

We examined age, sex, previous ATI consultations and antibiotic history prior to tonsillectomy. Prior ATI consultation frequency was a proxy measure for the severity of ATI infection preceding tonsillectomy. We also examined a number of co-morbid conditions, which are potentially associated with increased risk of URTI and tonsillectomy: asthma, Down's syndrome, cardiac disorders including congenital heart defects, immuno-deficiency, diabetes mellitus, as well as previous inhaled corticosteroid use. [3] [14] We studied practice-level deprivation using 'Index of Multiple Deprivation' scores based on the location of the general practice.

Statistical analysis

We used Fisher's exact test to investigate the differences in the baseline categorical characteristics of children in both study groups and the unpaired t-test to compare continuous variables. We calculated the mean (with 95% confidence intervals) for ATI consultations per child for both groups at: baseline (1998-2001), 1-3 years post-index date (2002-2004) and 4-6 years post-index date (2005-2007), in those with sufficient follow-up. We used the paired t-test to compare the mean differences within each group, followed by

For peer review only - http://bmjopen.bmj.com/site/about/guidelines.xhtml

the unpaired t-test to compare the mean differences between the two groups. A 2-sided P value of <0.05 was considered to be statistically significant.

Finally, we used logistic regression to investigate the outcomes of: ≥3 versus <3 ATI consultations, in 2002-2004 and 2005-2007 compared with the baseline in 1998-2001, after adjusting for potential confounders. This binary cut-off in ATI consultation frequency was selected to potentially approximate to at least one ATI consultation per year post-index date, respectively, in both time periods. We calculated crude odds ratios (OR) followed by adjusted OR, after controlling for the following potential confounders: age, sex, the co-morbid conditions mentioned above including at least one prior corticosteroid inhaler prescription, prior ATI consultations, prior antibiotics, practice-level deprivation and clustering of general practices. We tested for an interaction between age and sex for tonsillectomy *a priori* based on the observations from previous studies.[2, 15] There were no missing data for the socio-demographic variables (age, sex and practice deprivation score). However, the completeness levels of data for the passive smoking and body mass index variables were very poor (<10%), so we excluded these variables and did not adjust for them in the logistic regresssion analysis. Analysis was performed using Stata/SE version 11.1.[16]

For peer review only - http://bmjopen.bmj.com/site/about/guidelines.xhtml

RESULTS

For the tonsillectomy group, there were 450 children with at least 3 years of prior registration history and ≤3 ATI consultations who underwent tonsillectomy in 2001 (index date). Among these children, 92% (414/450) also had 3 years' follow-up and 80% (358/450) had 6 years' follow-up post-index date, respectively. For the comparison group, there were 13442 children with at least 3 years of prior history and ≤3 ATI consultations. Among these children, 90% (12053/13442) also had 3 years' follow-up and 79% (10593/13442) had 6 years' follow-up post-index date, respectively.

Among the 450 children who underwent tonsillectomy with ≤3 ATI consultations at baseline, the highest percentage of tonsillectomies were performed on children aged 4-7 years (46%, 209/450) and 53% (237/450) were performed on girls.

Children aged 4-15 years in the tonsillectomy group had a higher mean ATI consultation rate (1.3 over 3 years) than those in the comparison group (0.4 over 3 years) at baseline, 1998-2001 (Table 1). The mean number of baseline antibiotic prescriptions was also higher in the tonsillectomy group (3.7 and 2.1 prescriptions over the 3 years) in the tonsillectomy and comparison groups, respectively, Table 1).

For peer review only - http://bmjopen.bmj.com/site/about/guidelines.xhtml

Table 1: Characteristics of the study population at baseline (1998-2001) in the tonsillectomy and no tonsillectomy (comparison) groups

	Tonsillectomy ≤3 ATI consultations (N=450)		No Tonsillectomy ≤3 ATI consultations (N=13442)		
					**t-test**
**Mean (SD) Age**	8.6(3.4)		9.3(3.6)		**P<0.001**
**Mean (SD) no. ATI consultations in 3 years prior to index date**	1.3(1.1)		0.4(0.8)		**P<0.001**
**Mean (SD) no. of antibiotics in 3 years prior to index date**	3.7(3.9)		2.1(2.7)		**P<0.001**
	**n**	**%**	**n**	**%**	**χ^2 test**
**Sex**					
**girls**	237	53	7285	54	P=0.52
**boys**	213	47	6157	46	
**Deprivation quintile**					
**1 (least deprived)**	105	23	2848	21	P=0.10
**5 (most deprived)**	103	23	3545	26	
**COMORBIDITIES**					
**asthma**	91	20	2302	17	P=0.10
**cardiac disease and defects**	2	0	11	0	P=0.07
**Down's syndrome**	3	1	15	0	**P=0.02**
**immunodeficiency**	0	0	1	0	P=1.00
**diabetes**	6	1	239	2	P=0.59
**≥ 1 corticosteroid inhaler prescription**	120	27	3259	24	P=0.24

P values shown in bold where statistically significant

ATI – acute throat infection

SD – standard deviation

For peer review only - http://bmjopen.bmj.com/site/about/guidelines.xhtml

In the tonsillectomy group, the 3-year mean ATI consultation rate among children aged 4-15 years decreased from 1.31 to 0.66 between 1998-2001 and 2002-2004, and was 0.60 in 2005-2007 (Table 2). This represented a difference in mean ATI consultation rate of -0.65 (95% CI -0.78 to -0.51) between 1998-2001 and 2002-2004, and then of -0.72 (95% CI -0.88 to -0.56) in 2005-2007 compared with the same baseline (1998-2001).

By contrast, in the comparison group, the 3-year mean ATI consultation rate increased from 0.44 to 2.25 between 1998-2001 and 2002-2004, and was 0.93 in 2005-2007 (Table 2). This represented a difference in mean ATI consultation rate of +1.81 (95% CI 1.78 to 1.84) between 1998-2001 and 2002-2004, and then of +0.49 (95% CI 0.46 to 0.52) in 2005-2007 compared with 1998-2001.

Among children aged 4-15 years, comparing those who underwent tonsillectomy with those who did not, the unpaired t-test showed a net reduction in 3-year mean ATI consultations per child of 2.46 (95% CI 2.29 to 2.63, P<0.001) over 1-3 years' follow-up and of 1.21 (95% CI 1.04 to 1.38, P<0.001) over 4-6 years' follow-up, compared with baseline. This represents a net reduction of 3.67 ATI consultations over 6 years' follow-up or approximates to an annual reduction in mean ATI consultations of 0.61 per child, per year, over 6 years' follow-up.

For peer review only - http://bmjopen.bmj.com/site/about/guidelines.xhtml

Table 2 : Mean differences in acute throat infection (ATI) consultations per child prior to and subsequent to the index date in 2001

	mean number of ATI consultations and mean differences (with 95% CIs) comparing 3 year time intervals (1998-2001, 2002-2004 and 2005-2007)							
	n	1998-2001 3 years prior to index	2002- 2004 1-3 years post index	mean difference between 1998-2001 and 2002-2004 &	n	1998-2001 3 years prior to index	2005-2007 4-6 years post index	mean difference between 1998-2001 and 2005-2007&
	3 years prior to index date and 1-3 years follow up				3 years prior to index date and 4-6 years follow up			
**ALL CHILDREN**								
No Tonsillectomy	12053	0.44	2.25	**+1.81 (1.78 to 1.84)** ***	10593	0.44	0.93	**+0.49 (0.46** to **0.52)***
Tonsillectomy	414	1.31	0.66	**-0.65 (-0.78 to -0.51)***	358	1.32	0.6	**-0.72 (-0.88 to -0.56)***
**GIRLS 4-7 years**								
No Tonsillectomy	2375	0.7	2.43	**+1.74 (1.66 to 1.81)***	2092	0.7	1.04	**+0.35 (0.27 to 0.42)***
Tonsillectomy	78	1.32	0.76	**-0.56 (-0.94 to -0.19)**	71	1.34	0.65	**-0.69 (-1.08 to -0.31)***
**BOYS 4-7 years**								
No Tonsillectomy	2242	0.7	2.29	**+1.59 (1.51 to 1.67)***	1989	0.69	0.76	+0.06 (0.00 to 0.13)
Tonsillectomy	110	1.32	0.83	**-0.49 (-0.81 to -0.18)**	92	1.32	0.55	**-0.76 (-1.02 to -0.50)***
**GIRLS 8-11 years**								
No Tonsillectomy	1944	0.35	2.23	**+1.87 (1.80 to 1.95)***	1744	0.35	1.08	**+0.73 (0.65 to 0.81)***
Tonsillectomy	71	1.43	0.73	**-0.70 (-0.99 to -0.42)***	65	1.43	0.92	**-0.51 (-1.00 to -0.01)***
**BOYS 8-11 years**								
No Tonsillectomy	1526	0.31	2.04	**+1.73 (1.65 to 1.80)***	1395	0.31	0.71	**+0.40 (0.33 to 0.47)***
Tonsillectomy	59	1.17	0.34	**-0.83 (-1.16 to -0.51)***	49	1.22	0.39	**-0.84 (-1.23 to -0.45)***
**GIRLS 12-15 years**								
No Tonsillectomy	2206	0.23	2.45	**+2.22 (2.14 to 2.30)***	1866	0.23	1.2	**+0.97 (0.89 to 1.04)***
Tonsillectomy	66	1.29	0.62	**-0.67 (-0.95 to -0.38)***	55	1.22	0.62	**-0.60 (-0.96 to -0.24)**
**BOYS 12-15 years**								
No Tonsillectomy	1760	0.2	1.91	**+1.70 (1.64 to 1.77)***	1507	0.21	0.68	**+0.47 (0.41 to 0.53)***
Tonsillectomy	30	1.27	0.37	**-0.90 (-1.28 to -0.52)***	26	1.42	0.23	**-1.19 (-1.69 to -0.69)***

& paired t-test
* P <0.05
** P<0.01
*** P<0.001

For peer review only - http://bmjopen.bmj.com/site/about/guidelines.xhtml

The crude odds ratio (OR) for the effect of tonsillectomy on the outcome of ≥3 ATI consultations over 1-3 years' follow-up was 0.18 (95% CI: 0.13-0.27) and the adjusted OR was 0.12 (95% CI: 0.08-0.17). The crude OR for ≥3 ATI consultations over 4-6 years' follow-up was 0.36 (95% CI: 0.22-0.59), whilst the adjusted OR was 0.24 (95% CI: 0.14-0.41). The crude odds ratios were adjusted for age, sex, the co-morbid conditions mentioned earlier, including at least one prior corticosteroid inhaler prescription, prior ATI consultations, prior antibiotics, practice-level deprivation and clustering of general practices. Prior ATI consultations, followed by prior antibiotics, were the strongest confounders of the association between tonsillectomy and the outcome of ATI consultations.

DISCUSSION

Main findings

Children aged 4-15 years with a history of low ATI consultation frequency at baseline (≤3 ATI consultations during three years) who underwent tonsillectomy experienced a net reduction in the mean ATI consultation rate of 3.67 over six years' follow up, representing 2.46 (95% CI 2.29 to 2.63, P<0.001) over the first three years and 1.21 (95% CI 1.04 to 1.38, P<0.001) per child over the subsequent three years' follow-up, compared with those who did not undergo surgery. This approximates to an annual reduction in the mean ATI consultation rate of 0.61 per child, per year, over six years' follow-up. Tonsillectomy was also associated with much lower odds of ≥3 ATI consultations throughout the six year follow-up period.

For peer review only - http://bmjopen.bmj.com/site/about/guidelines.xhtml

Strengths and limitations of the study

This is among the first studies to explore the longer-term impact of tonsillectomy in a general practice setting in the UK, covering nine years. We analysed ATI consultation data for up to six years' follow-up in both groups and, to our knowledge, such a long follow-up period has not been studied before. We used routine individual-level general practice data from a nationally representative population-based dataset, which limited response bias. The large number of comparisons from a wide population base increased the power of the study.

However, there are also a number of limitations to our study. Firstly, our measure of ATI frequency relied on visits to the general practitioner rather than self-reporting. Although this is an objective measure, we recognise it relies on health-seeking behaviour (HSB) and could, for example, underestimate the frequency of ATI episodes since not every child attends with every ATI episode. Secondly, although we endeavoured to exclude all children who may have undergone tonsillectomy for the SDB spectrum, we are unlikely to have captured everyone who had surgery for this indication. Thirdly, the losses to follow-up could have created attrition bias, but this is difficult to minimise and this also affects experimental studies. The losses to follow-up were similar in the tonsillectomy and comparison groups over the three and six year follow-up periods and were both under 20% at six years' follow-up. Fourthly, there were higher baseline ATI consultation and antibiotic means in the tonsillectomy group. However, we adjusted for prior ATI consultations and antibiotics which made the two groups more comparable. Finally, the large size of the study population with demographic and co-morbidity variables, which were available from the CPRD dataset, allowed us to adjust for a number of potential confounding factors. However, as with most

For peer review only - http://bmjopen.bmj.com/site/about/guidelines.xhtml

retrospective cohort studies, we were unable to study all known and unknown potential confounding variables and thus there could be residual confounding, which we could not control for.

Comparisons with other studies

A Dutch RCT among children aged 2-8 years who did not fulfil the Paradise criteria [4] for severe recurrent throat infection frequency found the effects of tonsillectomy were more pronounced in children who had 3-6 throat infections in the year prior to entering the trial than in those with ≤2 throat infections.[8] Among children in this latter group, the authors reported fewer throat infections (-0.21, 95% CI -0.06 to -0.36) and fewer sore throats (-0.60, 95% CI -0.30 to -0.90) per person year in the tonsillectomy group but concluded that these differences were not large enough to be clinically significant, although the median follow-up time was only 22 months. [8] Although ours was a cohort study, we similarly found that although tonsillectomy led to a statistically significant reduction in ATI consultation rates among children with low baseline ATI, this was not necessarily clinically significant.

A UK study which analysed CPRD data for annual sore throat consultation rates found that the rates per 1000 registered patients declined by 50% between 1995 and 2000 among children aged 5-16 years.[17] The authors reported sore throat consultation rates in 2000 of 59 and 76 per 1000 registered patients among children aged 5-10 and 11-16 years, respectively. However, that study did not investigate the annual consultation rates for an individual child. The authors proposed that the overall decline in consultation rates may reflect that patients were increasingly self-managing minor illnesses. [17]

For peer review only - http://bmjopen.bmj.com/site/about/guidelines.xhtml

We found the natural history among children of all ages who did not have tonsillectomy was for a gradual increase in the number of ATI consultations. This is contrary to previous studies, which reported that the natural history of sore throats is to attenuate over time.[18] [19, 20] However, our study was based in a community setting and focused on a population of children with low baseline ATI who may have emerging natural history whereas other studies have selected children with higher baseline ATI from the outset. If we had followed children up for longer we may have observed a decline over time.

Clinical implications of the study

Overall, children aged 4-15 years, who underwent tonsillectomy compared with those who did not, experienced an 88% and 76% reduced risk of having ≥3 ATI consultations over 1-3 years and 4-6 years' follow-up, respectively. However, the observed decline in ATI consultations might also, in part, be attributed to adeno-tonsillectomy operations reducing rhino-sinusitis or otitis media infections, which were coded as URTIs.[21, 22] [23]

There was no evidence of an association between tonsillectomy and deprivation level, which is in contrast to some recent studies and reports.[24-27] However, we only had practice-level deprivation data and not individual-level data, which could account for the absence of an association.

Children in our study who underwent tonsillectomy in 2001 had ≤3 ATI consultations documented in general practice during the three years prior to their operation. This is

For peer review only - http://bmjopen.bmj.com/site/about/guidelines.xhtml

markedly less than the minimum threshold recommended by national guidelines, reviews and the Paradise criteria for frequency of episodes prior to tonsillectomy. [1] [4] [20] [28] [29] These findings may reflect lower thresholds being followed or perhaps represent sub-optimal documentation of sore throat consultations prior to tonsillectomy in primary care settings. Although we assumed that parents would have consulted if the ATI was severe, the low consultation rate could reflect HSB, whereby some parents may not present with ATI episodes if, for example, antibiotics were not issued at prior consultations. However, this depends on the individual GP's attitudes and threshold for prescribing antibiotics for ATIs.

Other possible explanations for these low ATI consultation rates are that we did not have access to the 'free text' within individual medical consultation records, which may contain additional details about ATI episodes. Children may have attended with concurrent signs or symptoms and so other diagnostic codes, such as otitis media or specific viral URTIs, may have been recorded with details of the sore throat symptoms included within the 'free text'. As previously mentioned, overall annual consultation rates for sore throat declined by 50% among children aged 5-16 years between 1995 and 2000 in the UK, which may further account for our lower ATI consultation rates at baseline.[17] We did not have consultations from other primary care settings, direct Accident and Emergency department attendances or hospital admissions, which have been increasing over recent years and may not be well-documented within general practice records.[30] Therefore, we interpret our findings with the caveat that there could be other possible explanations for the low documentation rate of ATI consultations. However, the strength of our study is that we are consistent in comparing the ATI consultation frequency prior to and subsequent to the index date for each child in a particular general practice, which is where the vast majority of consultations

For peer review only - http://bmjopen.bmj.com/site/about/guidelines.xhtml

with children occur.[10] We recommend future studies should examine individual consultations in greater detail, as well as attendances in community and hospital emergency settings, to attempt to capture ATI consultations within different healthcare settings.

Our findings suggest that there is limited clinical benefit in performing tonsillectomy for children with low baseline ATI consultation rates. This reinforces existing guidance that only children with severe throat infection disease at baseline should be referred for tonsillectomy. Previous research has suggested that the operation is beneficial in the short-term for those with more serious ATI morbidity at baseline; although its benefit in the longer-term is still not clear and this still needs to be investigated. Tonsillectomy is a major operation so it is important that surgery confers significant individual and societal benefits, including fewer days off work by parents affected by sick children.

Our study also highlights the importance of diligent documentation of ATI consultations within general practice. There is a need to validate diagnostic screening tools among children, such as the Centor criteria and McIsaac scores, for group A β-haemolytic streptococcal throat infections. This may help towards more accurate diagnosis, recording and more appropriate management of ATI consultations in general practice.

Conclusion

Our study found a statistically significant reduction in ATI consultation rates among children with low baseline ATI consultation rates who underwent tonsillectomy compared with those who did not. However, for this subgroup of children, the relatively modest reduction in ATI consultation rates in clinical terms may be outweighed by the potential risks related to the

For peer review only - http://bmjopen.bmj.com/site/about/guidelines.xhtml

operation. The long-term clinical effectiveness of tonsillectomy for children, across the ATI frequency spectrum, still needs to be evaluated. This needs to be undertaken in conjunction with updated cost-effectiveness models, stratified by age, sex and baseline ATI rates, so that clinicians can risk profile children to determine which subgroups of children are most likely to benefit from surgery.

For peer review only

For peer review only - http://bmjopen.bmj.com/site/about/guidelines.xhtml

FUNDING: EK is funded by a National Institute for Health Research Doctoral Research Fellowship (2009-02-78). SS is funded by a a National Institute for Health Research Career Development Fellowship (NIHR CDF-2011-04-048). HW is funded by NIHR Research Design Service.This article presents independent research commissioned by the National Institute for Health Research (NIHR) under the Collaborations for Leadership in The views expressed in this publication are those of the author(s) and not necessarily those of the NHS, the NIHR or the Department of Health.

ETHICAL APPROVAL : The study was approved by the MHRA Independent Scientific Advisory Committee on Database Research

ACKNOWLEDGEMENTS: Data was obtained from the Clinical Practice Research Datalink, CPRD (formerly known as the General Practice Research Database). The CPRD is jointly funded by the NHS National Institute for Health Research (NIHR) and the Medicines and Healthcare products Regulatory Agency (MHRA).

COMPETING INTERESTS: none declared

DATA SHARING STATEMENT: no additional data are available

CONTRIBUTOR STATEMENT: The study was conceived by EK, SS and MS. The study was designed by EK, SS, HW and AB. VC extracted the data. EK analysed the data. HW provided statistical advice. EK wrote the first draft of the manuscript. All authors contributed to the interpretation of the findings and revised the paper critically for intellectual content. All authors have seen and approved this version of the manuscript. EK and SS are the guarantors of this study.

For peer review only - http://bmjopen.bmj.com/site/about/guidelines.xhtml

REFERENCES

1. SIGN. Scottish Intercollegiate Guidelines Network 117. Management of sore throat and indications for tonsillectomy. . April 2010. http://www.sign.ac.uk/pdf/sign117.pdf.

2. Lock C, Wilson J, Steen N, et al. North of England and Scotland Study of Tonsillectomy and Adeno-tonsillectomy in Children(NESSTAC): a pragmatic randomised controlled trial with a parallel non-randomised preference study. Health Technol Assess. 2010 Mar;14(13):1-164, iii-iv.

3. Orvidas LJ, St Sauver JL, Weaver AL. Efficacy of tonsillectomy in treatment of recurrent group A beta-hemolytic streptococcal pharyngitis. Laryngoscope. 2006 Nov;116(11):1946-50.

4. Paradise JL, Bluestone CD, Bachman RZ, et al. Efficacy of tonsillectomy for recurrent throat infection in severely affected children. Results of parallel randomized and nonrandomized clinical trials. N Engl J Med. 1984 Mar 15;310(11):674-83.

5. Koshy E, Curcin V, Bottle A, et al. Sore throat consultations in general practice prior to tonsillectomy among eight hundred and sixty-three children in England: is this in accordance with the SIGN guidelines? Clin Otolaryngol. 2013 Jun;38(3):266-70.

6. Toh A, Mullin A, Grainger J, et al. Indications for tonsillectomy: are we documenting them? Annals of the Royal College of Surgeons of England. 2009 Nov;91(8):697-9.

For peer review only - http://bmjopen.bmj.com/site/about/guidelines.xhtml

7. Paradise JL, Bluestone CD, Colborn DK, et al. Tonsillectomy and adenotonsillectomy for recurrent throat infection in moderately affected children. Pediatrics. 2002 Jul;110(1 Pt 1):7-15.

8. van Staaij BK, van den Akker EH, Rovers MM, et al. Effectiveness of adenotonsillectomy in children with mild symptoms of throat infections or adenotonsillar hypertrophy: open, randomised controlled trial. BMJ. 2004 Sep 18;329(7467):651.

9. Tsou YA, Lin CC, Lai CH, et al. Does Adenotonsillectomy really reduced clinic visits for pediatric upper respiratory tract infections? A national database study in Taiwan. Int J Pediatr Otorhinolaryngol. 2013 May;77(5):677-81.

10. Hippisley-Cox J, Vinogradova Y. Trends in Consultation Rates in General Practice 1995/1996 to 2008/2009: Analysis of the QResearch® database. http://www.ic.nhs.uk/webfiles/publications/gp/Trends_in_Consultation_Rates_in_General_Practice_1995_96_to_2008_09.pdf. 2009.

11. Saxena S, Francis N, Sharland M. Primary care of children: the unique role of GPs. Br J Gen Pract. 2012 Jul;62(600):340-1.

12. CPRD. Clinical Practice Research Datalink. http://www.cprd.com/.

13. Parker NP, Walner DL. Trends in the indications for pediatric tonsillectomy or adenotonsillectomy. Int J Pediatr Otorhinolaryngol. 2011 Feb;75(2):282-5.

For peer review only - http://bmjopen.bmj.com/site/about/guidelines.xhtml

14. Ram G, Chinen J. Infections and immunodeficiency in Down syndrome. Clinical and experimental immunology. 2011 Apr;164(1):9-16.

15. Vestergaard H, Wohlfahrt J, Westergaard T, et al. Incidence of tonsillectomy in Denmark, 1980 to 2001. Pediatr Infect Dis J. 2007 Dec;26(12):1117-21.

16. Stata/SE version 11.1 http://www.stata.com/stata11/point1.html.

17. Ashworth M, Charlton J, Latinovic R, et al. Age-related changes in consultations and antibiotic prescribing for acute respiratory infections, 1995-2000. Data from the UK General Practice Research Database. Journal of clinical pharmacy and therapeutics. 2006 Oct;31(5):461-7.

18. Buskens E, van Staaij B, van den Akker J, et al. Adenotonsillectomy or watchful waiting in patients with mild to moderate symptoms of throat infections or adenotonsillar hypertrophy: a randomized comparison of costs and effects. Arch Otolaryngol Head Neck Surg. 2007 Nov;133(11):1083-8.

19. van Staaji BK, van den Akker EH, Rovers MM, et al. Effectiveness of adenotonsillectomy in children with mild symptoms of throat infections or adenotonsillar hypertrophy: open, randomised controlled trial. Clin Otolaryngol. 2005 Feb;30(1):60-3.

20. Burton MJ, Glasziou PP. Tonsillectomy or adeno-tonsillectomy versus non-surgical treatment for chronic/recurrent acute tonsillitis. Cochrane Database Syst Rev. 2009(1):CD001802.

For peer review only

For peer review only - http://bmjopen.bmj.com/site/about/guidelines.xhtml

21. van den Aardweg MT, Schilder AG, Herkert E, et al. Adenoidectomy for otitis media in children. Cochrane Database Syst Rev. 2010(1):CD007810.

22. Paradise JL, Bluestone CD, Colborn DK, et al. Adenoidectomy and adenotonsillectomy for recurrent acute otitis media: parallel randomized clinical trials in children not previously treated with tympanostomy tubes. JAMA. 1999 Sep 8;282(10):945-53.

23. Darrow DH, Siemens C. Indications for tonsillectomy and adenoidectomy. Laryngoscope. 2002 Aug;112(8 Pt 2 Suppl 100):6-10.

24. Bisset AF, Russell D. Grommets, tonsillectomies, and deprivation in Scotland. BMJ. 1994 Apr 30;308(6937):1129-32.

25. Croxford R, Friedberg J, Coyte PC. Socio-economic status and surgery in children: myringotomies and tonsillectomies in Ontario, Canada, 1996-2000. Acta paediatrica (Oslo, Norway : 1992). 2004 Sep;93(9):1245-50.

26. Koshy E, Bottle A, Murray J, et al. Changing indications and socio-demographic determinants of (adeno)tonsillectomy among children in England--are they linked? A retrospective analysis of hospital data. PLoS One. 2014;9(8):e103600.

27. Chief Medical Officer's Annual Report (2005) Department of Health. http://www.dh.gov.uk/prod_consum_dh/groups/dh_digitalassets/@dh/@en/documents/digitalasset/dh_4137367.pdf.

For peer review only - http://bmjopen.bmj.com/site/about/guidelines.xhtml

28. Baugh RF, Archer SM, Mitchell RB, et al. Clinical practice guideline: tonsillectomy in children. Otolaryngol Head Neck Surg. 2011 Jan;144(1 Suppl):S1-30.

29. SIGN. Scottish Intercollegiate Guidelines Network 34: Management of sore throat and indications for tonsillectomy. Available on specific request from SIGN, as it is no longer directly accessible from the SIGN website. (1999) http://www.atb.healthnet.sk/Sore_Throat/sign34-sore-throat.pdf (accessed 15th October 2014).

30. Koshy E, Murray J, Bottle A, et al. Significantly increasing hospital admissions for acute throat infections among children in England: is this related to tonsillectomy rates? Arch Dis Child. 2012 Dec;97(12):1064-8.

For peer review only - http://bmjopen.bmj.com/site/about/guidelines.xhtml

Tonsillectomy among children with low baseline acute throat infection consultation rates in UK general practices: a cohort study

Elizabeth Koshy,[1] Hilary Watt,[1] Vasa Curcin,[12] Alex Bottle,[1] Mike Sharland[32] Sonia Saxena[1]

Elizabeth Koshy, MRCGP
Hilary Watt, MSc CStat
Vasa Curcin, PhD
Alex Bottle, PhD
Mike Sharland, FRCPCH
Sonia Saxena, FRCGP

[1] Department of Primary Care and Public Health, Imperial College London
[2] Department of Primary Care and Public Health Sciences, Kings College London
[32] Paediatric Infectious Diseases Unit, St George's Hospital NHS Trust, London

Paediatric Infectious Diseases Research Group, St George's University London, London

Keywords: tonsillectomy, tonsillitis, child, otorhinolaryngologic diseases, throat

Corresponding author:

Dr. Elizabeth Koshy
Department of Primary Care and Public Health, Imperial College London,
London W6 8RP
Email:e.koshy@imperial.ac.uk

For peer review only

For peer review only - http://bmjopen.bmj.com/site/about/guidelines.xhtml

ABSTRACT

Objective: To investigate the effectiveness of tonsillectomy in reducing acute throat infection (ATI) consultation rates over 6 years' follow-up among children with low baseline ATI consultation rates.

Design: Retrospective cohort study

Setting: UK general practices from the Clinical Practice Research Datalink.

Participants: Children aged 4-15 years with ≤3 ATI consultations during the 3 years prior to 2001 (baseline). 450 children who underwent tonsillectomy (tonsillectomy group) and 13,442 other children with an ATI consultation (comparison group) in 2001.

Main outcome measures: Mean differences in ATI consultation rates over the first 3 years' and subsequent 3 years' follow-up compared with 3 years prior to 2001 (baseline); odds of ≥3 ATI consultations at the same time points.

Results: Among children in the tonsillectomy group, the 3-year mean ATI consultation rate decreased from 1.31 to 0.66 over the first 3 years' follow-up and further declined to 0.60 over the subsequent 3 years' follow-up period. Compared with children who had no operation, those who underwent tonsillectomy experienced a reduction in 3-year mean ATI consultations per child of 2.5 (95% CI 2.3-2.6, P<0.001) over the first 3 years' follow-up, but

For peer review only

For peer review only - http://bmjopen.bmj.com/site/about/guidelines.xhtml

only 1.2 (95% CI 1.0-1.4, P<0.001) over the subsequent 3 years' follow-up compared with baseline, respectively. This equates to a mean reduction of 3.7 ATI consultations over a six year period and approximates to a mean annual reduction of 0.6 ATI consultations per child, per year, over 6 years' follow-up. Children who underwent tonsillectomy were also much less likely to experience ≥3 ATI consultations during the first 3 years' follow-up (adjusted OR=0.12, 95% CI:0.08-0.17) and the subsequent 3 years' follow-up (adjusted OR=0.24, 95% CI:0.14-0.41).

Conclusion: Among children with low baseline ATI rates, there was a statistically significant reduction in ATI consultation rates over 6 years' follow-up. However, the relatively modest clinical benefit needs to be weighed against the potential risks and complications associated with surgery.

For peer review only

Strengths and limitations of study

- This large cohort study is among the first studies to explore the longer-term impact of tonsillectomy among children with low baseline acute throat infection (ATI) consultation rates in a general practice setting in the United Kingdom. We examined ATI consultation history over a 6 year follow-up period using routine, patient-level general practice data from a nationally representative population-based dataset, which limited response bias.

- The main limitations relate to the diagnostic coding of ATIs by clinicians in general practice and that attendance for acute throat infection consultations isare influenced by the health-seeking behaviour of parents and the management of ATIs by general practitioners.

For peer review only

INTRODUCTION

Tonsillectomy is one of the most common operations performed in children and the majority are performed for frequent acute tonsillitis episodes, for which there is evidence of benefit.[1, 2] [3] [4] However, differentiating between acute tonsillitis, pharyngitis and other causes of acute throat infections (ATI) is challenging in a general practice setting. Children who only have a record of low frequency ATI rates can undergo the operation, despite a lack of evidence for its clinical effectiveness in this group. [1] [5-8] Therefore, in children with low baseline frequency of ATI, clarity is needed about the potential benefits of tonsillectomy over time. Randomised controlled trials (RCT) and previous observational studies have typically not looked beyond two or three years.[3, 4, 7] [8, 9] It is difficult to justify the costs, ethics and logistical problems of mounting new trials over an extended period of time to address this gap in the literature.

The natural history of ATI in children is to spontaneously resolve.[1, 8] This can make it difficult to assess whether tonsillectomy has been effective or whether the ATI frequency would have attenuated over time. Therefore, it is important to compare the prior and subsequent ATI history among children who undergo tonsillectomy with those who do not to evaluate its impact over an extended period of time. Information about the medium and longer-term benefits of tonsillectomy on key outcomes including reduction in subsequent ATI is important for clinicians and to aid parental decision-making. About 98% of children are registered with a general practice in the UK and the vast majority of consultations occur in this setting, which enables real-time ATI consultations to be studied over time.[10, 11]

For peer review only - http://bmjopen.bmj.com/site/about/guidelines.xhtml

Our aim was to investigate whether children with low baseline ATI consultation rates who underwent tonsillectomy experienced a significant reduction in subsequent ATI consultation rates compared with children who did not undergo surgery over six years' follow-up, using longitudinal data from the Clinical Practice Research Datalink (CPRD) database.

MATERIALS and METHODS

Data source

We used data from the CPRD, which contains individual-level computerised medical records from about 600 general practices and covers data for about 8% of the UK population. [12] The data include demographic details, prescriptions and clinical events, which are entered as diagnostic Read codes by general practitioners during the routine clinical management of patients. CPRD has been extensively used for research.[12]

Study design, ATI definition and study population

We conducted a cohort study of children aged 4-15 years who had ≤<3 ATI consultations at baseline. Children with a record of tonsillectomy or adeno-tonsillectomy between 1st January and 31st December 2001 formed the tonsillectomy group. The comparison group comprised of children with no history of tonsillectomy but a record of a sore throat consultation between these dates (supplementary figure S1). The date for the tonsillectomy record or sore throat consultation in 2001 represented the index date. The study period spanned nine years in total, comprising three years prior to the index date (baseline) and six years subsequent to the index date. The period of follow-up for subsequent consultations was from the index date, which was the date of the first tonsillectomy record or sore throat consultation in 2001, to the date of censoring (the latest CPRD data collection, end of the

For peer review only

For peer review only - http://bmjopen.bmj.com/site/about/guidelines.xhtml

study period of six years post-index date or the patient's transfer out of the practice, whichever came first). We studied ATI consultations prior to and subsequent to the index date.

We developed a list of Read codes (supplementary table S2) to define prior and subsequent ATI consultations, which encompassed sore throat symptoms and signs, tonsillitis, pharyngitis and also non-specific upper respiratory tract infection (URTI) codes used by general practitioners to record a throat infection consultation. We searched for non-specific URTI in addition to sore throat consultations, as our previous study showed that the yield can be low by searching exclusively for sore throat consultation record codes. [5]

To enable us to account for prior history of ATI, as a marker of severity, we included children aged 4-15 years if they had ≥3 years' registration prior to the index date (baseline) (supplementary figure S1). We focused our target population on children with low baseline ATIs (with a mild severity spectrum) by excluding those with >3 ATI consultations during the baseline period (1998-2001). Children with a history of snoring, hypertrophied tonsils, sleep apnoea, polysomnography or obstructive sleep apnoea were also excluded from both groups, as this is a different indication for tonsillectomy and our focus was to examine decision-making about tonsillectomy for ATI, not the sleep-disordered breathing (SDB) spectrum. Children aged <4 years were excluded, as pre-school children are more commonly referred for the SDB spectrum than for recurrent ATI.[13] The final study population consisted of 450 children in the tonsillectomy group and 13442 children in the comparison group (supplementary figure S1).

For peer review only

Outcome measures

Our main outcome was the mean ATI consultation rate per child compared over three time frames across the nine year study period (1998-2007): three years prior to the index date (1998-2001), which is used as a baseline comparator in the main analyses, 1-3 years' follow-up post-index date (2002-2004) and 4-6 years' follow-up post-index date (2005-2007) (Table 2). We also calculated the odds of ≥3 versus <3 ATI consultations per child during the same follow-up time frames.

Potential risk factors

We examined age, sex, previous ATI consultations and antibiotic history prior to tonsillectomy. Prior ATI consultation frequency was a proxy measure for the severity of ATI infection preceding tonsillectomy. We also examined a number of co-morbid conditions, which are potentially associated with increased risk of URTI and tonsillectomy: asthma, Down's syndrome, cardiac disorders including congenital heart defects, immuno-deficiency, diabetes mellitus, as well as previous inhaled corticosteroid use. [3] [14] We studied practice-level deprivation using 'Index of Multiple Deprivation' scores based on the location of the general practice.

Statistical analysis

We used Fisher's exact test to investigate the differences in the baseline categorical characteristics of children in both study groups and the unpaired t-test to compare continuous variables. We calculated the mean (with 95% confidence intervals) for ATI consultations per child for both groups at: baseline (1998-2001), 1-3 years post-index date (2002-2004) and 4-6 years post-index date (2005-2007), in those with sufficient follow-up. We used the paired t-test to compare the mean differences within each group, followed by

For peer review only - http://bmjopen.bmj.com/site/about/guidelines.xhtml

the unpaired t-test to compare the mean differences between the two groups. A 2-sided P value of <0.05 was considered to be statistically significant.

Finally, we used logistic regression to investigate the outcomes of: ≥3 versus <3 ATI consultations, in 2002-2004 and 2005-2007 compared with the baseline in 1998-2001, after adjusting for potential confounders. This binary cut-off in ATI consultation frequency was selected to potentially approximate to at least one ATI consultation per year post-index date, respectively, in both time periods. We calculated crude odds ratios (OR) followed by adjusted OR, after controlling for the following potential confounders: age, sex, the co-morbid conditions mentioned above including at least one prior corticosteroid inhaler prescription, prior ATI consultations, prior antibiotics, practice-level deprivation and clustering of general practices. We tested for an interaction between age and sex for tonsillectomy *a priori* based on the observations from previous studies.[2, 15] There were no missing data for the socio-demographic variables (age, sex and practice deprivation score). However, the completeness levels of data for the passive smoking and body mass index variables were very poor (<10%), so we excluded these variables and did not adjust for them in the logistic regresssion analysis. Analysis was performed using Stata/SE version 11.1.[16]

For peer review only

RESULTS

For the tonsillectomy group, there were 450 children with at least 3 years of prior registration history and ≤3 ATI consultations who underwent tonsillectomy in 2001 (index date). Among these children, 92% (414/450) also had 3 years' follow-up and 80% (358/450) had 6 years' follow-up post-index date, respectively. For the comparison group, there were 13442 children with at least 3 years of prior history and ≤3 ATI consultations. Among these children, 90% (12053/13442) also had 3 years' follow-up and 79% (10593/13442) had 6 years' follow-up post-index date, respectively.

Among the 450 children who underwent tonsillectomy with ≤3 ATI consultations at baseline, the highest percentage of tonsillectomies were performed on children aged 4-7 years (46%, 209/450) and 53% (237/450) were performed on girls.

Children aged 4-15 years in the tonsillectomy group had a higher mean ATI consultation rate (1.3 over 3 years) than those in the comparison group (0.4 over 3 years) at baseline, 1998-2001 (Table 1). The mean number of baseline antibiotic prescriptions was also higher in the tonsillectomy group (3.7 and 2.1 prescriptions over the 3 years) in the tonsillectomy and comparison groups, respectively, Table 1).

For peer review only - http://bmjopen.bmj.com/site/about/guidelines.xhtml

Table 1: Characteristics of the study population at baseline (1998-2001) in the tonsillectomy and no tonsillectomy (comparison) groups

	Tonsillectomy ≤3 ATI consultations (N=450)		No Tonsillectomy ≤3 ATI consultations (N=13442)		
					**t-test**
**Mean (SD) Age**	8.6(3.4)		9.3(3.6)		**P<0.001**
**Mean (SD) no. ATI consultations in 3 years prior to index date**	1.3(1.1)		0.4(0.8)		**P<0.001**
**Mean (SD) no. of antibiotics in 3 years prior to index date**	3.7(3.9)		2.1(2.7)		**P<0.001**
	**n**	**%**	**n**	**%**	**χ^2 test**
**Sex**					
**girls**	237	53	7285	54	P=0.52
**boys**	213	47	6157	46	
**Deprivation quintile**					
**1 (least deprived)**	105	23	2848	21	P=0.10
**5 (most deprived)**	103	23	3545	26	
**COMORBIDITIES**					
**asthma**	91	20	2302	17	P=0.10
**cardiac disease and defects**	2	0	11	0	P=0.07
**Down's syndrome**	3	1	15	0	**P=0.02**
**immunodeficiency**	0	0	1	0	P=1.00
**diabetes**	6	1	239	2	P=0.59
**≥ 1 corticosteroid inhaler prescription**	120	27	3259	24	P=0.24

P values shown in bold where statistically significant

ATI – acute throat infection

SD – standard deviation

For peer review only - http://bmjopen.bmj.com/site/about/guidelines.xhtml

In the tonsillectomy group, the 3-year mean ATI consultation rate among children aged 4-15 years decreased from 1.31 to 0.66 between 1998-2001 and 2002-2004, and was 0.60 in 2005-2007 (Table 2). This represented a difference in mean ATI consultation rate of -0.65 (95% CI -0.78 to -0.51) between 1998-2001 and 2002-2004, and then of -0.72 (95% CI -0.88 to -0.56) in 2005-2007 compared with the same baseline (1998-2001).

By contrast, in the comparison group, the 3-year mean ATI consultation rate increased from 0.44 to 2.25 between 1998-2001 and 2002-2004, and was 0.93 in 2005-2007 (Table 2). This represented a difference in mean ATI consultation rate of +1.81 (95% CI 1.78 to 1.84) between 1998-2001 and 2002-2004, and then of +0.49 (95% CI 0.46 to 0.52) in 2005-2007 compared with 1998-2001.

Among children aged 4-15 years, comparing those who underwent tonsillectomy with those who did not, the unpaired t-test showed a net reduction in 3-year mean ATI consultations per child of 2.46 (95% CI 2.29 to 2.63, P<0.001) over 1-3 years' follow-up and of 1.21 (95% CI 1.04 to 1.38, P<0.001) over 4-6 years' follow-up, compared with baseline. This represents a net reduction of 3.67 ATI consultations over 6 years' follow-up or approximates to an annual reduction in mean ATI consultations of 0.61 per child, per year, over 6 years' follow-up.

For peer review only - http://bmjopen.bmj.com/site/about/guidelines.xhtml

Table 2 : Mean differences in acute throat infection (ATI) consultations per child prior to and subsequent to the index date in 2001

	mean number of ATI consultations and mean differences (with 95% CIs) comparing 3 year time intervals (1998-2001, 2002-2004 and 2005-2007)							
	n	1998-2001 3 years prior to index	2002- 2004 1-3 years post index	mean difference between 1998-2001 and 2002-2004 &	n	1998-2001 3 years prior to index	2005-2007 4-6 years post index	mean difference between 1998-2001 and 2005-2007&
	3 years prior to index date and 1-3 years follow up				3 years prior to index date and 4-6 years follow up			
**ALL CHILDREN**								
No Tonsillectomy	12053	0.44	2.25	**+1.81 (1.78 to 1.84)*** **	10593	0.44	0.93	**+0.49 (0.46 to 0.52)*** **
Tonsillectomy	414	1.31	0.66	**-0.65 (-0.78 to -0.51)*** **	358	1.32	0.6	**-0.72 (-0.88 to -0.56)*** **
**GIRLS 4-7 years**								
No Tonsillectomy	2375	0.7	2.43	**+1.74 (1.66 to 1.81)*** **	2092	0.7	1.04	**+0.35 (0.27 to 0.42)*** **
Tonsillectomy	78	1.32	0.76	**-0.56 (-0.94 to -0.19)** **	71	1.34	0.65	**-0.69 (-1.08 to -0.31)*** **
**BOYS 4-7 years**								
No Tonsillectomy	2242	0.7	2.29	**+1.59 (1.51 to 1.67)*** **	1989	0.69	0.76	+0.06 (0.00 to 0.13)
Tonsillectomy	110	1.32	0.83	**-0.49 (-0.81 to -0.18)** **	92	1.32	0.55	**-0.76 (-1.02 to -0.50)*** **
**GIRLS 8-11 years**								
No Tonsillectomy	1944	0.35	2.23	**+1.87 (1.80 to 1.95)*** **	1744	0.35	1.08	**+0.73 (0.65 to 0.81)*** **
Tonsillectomy	71	1.43	0.73	**-0.70 (-0.99 to -0.42)*** **	65	1.43	0.92	**-0.51 (-1.00 to -0.01)* **
**BOYS 8-11 years**								
No Tonsillectomy	1526	0.31	2.04	**+1.73 (1.65 to 1.80)*** **	1395	0.31	0.71	**+0.40 (0.33 to 0.47)*** **
Tonsillectomy	59	1.17	0.34	**-0.83 (-1.16 to -0.51)*** **	49	1.22	0.39	**-0.84 (-1.23 to -0.45)*** **
**GIRLS 12-15 years**								
No Tonsillectomy	2206	0.23	2.45	**+2.22 (2.14 to 2.30)*** **	1866	0.23	1.2	**+0.97 (0.89 to 1.04)*** **
Tonsillectomy	66	1.29	0.62	**-0.67 (-0.95 to -0.38)*** **	55	1.22	0.62	**-0.60 (-0.96 to -0.24)** **
**BOYS 12-15 years**								
No Tonsillectomy	1760	0.2	1.91	**+1.70 (1.64 to 1.77)*** **	1507	0.21	0.68	**+0.47 (0.41 to 0.53)*** **
Tonsillectomy	30	1.27	0.37	**-0.90 (-1.28 to -0.52)*** **	26	1.42	0.23	**-1.19 (-1.69 to -0.69)*** **

& paired t-test
* P <0.05
** P<0.01
*** P<0.001

For peer review only - http://bmjopen.bmj.com/site/about/guidelines.xhtml

The crude odds ratio (OR) for the effect of tonsillectomy on the outcome of ≥3 ATI consultations over 1-3 years' follow-up was 0.18 (95% CI: 0.13-0.27) and the adjusted OR was 0.12 (95% CI: 0.08-0.17). The crude OR for ≥3 ATI consultations over 4-6 years' follow-up was 0.36 (95% CI: 0.22-0.59), whilst the adjusted OR was 0.24 (95% CI: 0.14-0.41). The crude odds ratios were adjusted for age, sex, the co-morbid conditions mentioned earlier, including at least one prior corticosteroid inhaler prescription, prior ATI consultations, prior antibiotics, practice-level deprivation and clustering of general practices. Prior ATI consultations, followed by prior antibiotics, were the strongest confounders of the association between tonsillectomy and the outcome of ATI consultations.

DISCUSSION

Main findings

Children aged 4-15 years with a history of low ATI consultation frequency at baseline (≤3 ATI consultations during three years) who underwent tonsillectomy experienced a net reduction in the mean ATI consultation rate of 3.67 over six years' follow up, representing 2.46 (95% CI 2.29 to 2.63, P<0.001) over the first three years and 1.21 (95% CI 1.04 to 1.38, P<0.001) per child over the subsequent three years' follow-up, compared with those who did not undergo surgery. This approximates to an annual reduction in the mean ATI consultation rate of 0.61 per child, per year, over six years' follow-up. Tonsillectomy was also associated with much lower odds of ≥3 ATI consultations throughout the six year follow-up period.

For peer review only - http://bmjopen.bmj.com/site/about/guidelines.xhtml

Strengths and limitations of the study

This is among the first studies to explore the longer-term impact of tonsillectomy in a general practice setting in the UK, covering nine years. We analysed ATI consultation data for up to six years' follow-up in both groups and, to our knowledge, such a long follow-up period has not been studied before. We used routine individual-level general practice data from a nationally representative population-based dataset, which limited response bias. The large number of comparisons from a wide population base increased the power of the study.

However, there are also a number of limitations to our study. Firstly, our measure of ATI frequency relied on visits to the general practitioner rather than self-reporting. Although this is an objective measure, we recognise it relies on health-seeking behaviour (HSB) and could, for example, underestimate the frequency of ATI episodes since not every child attends with every ATI episode. Secondly, although we endeavoured to exclude all children who may have undergone tonsillectomy for the SDB spectrum, we are unlikely to have captured everyone who had surgery for this indication. Thirdly, ~~the losses to follow-up were similar in the tonsillectomy and comparison groups over the three and six year follow-up periods and were both within 20% at six years' follow-up.~~ the ~~L~~losses to follow-up could have created attrition bias, but this is difficult to minimise and this also affects experimental studies. The losses to follow-up were similar in the tonsillectomy and comparison groups over the three and six year follow-up periods and were both under 20% at six years' follow-up. Fourthly, there were higher baseline ATI consultation and antibiotic means in the tonsillectomy group. However, we adjusted for prior ATI consultations and antibiotics which made the two groups more comparable. Finally, the large size of the study population with

For peer review only

For peer review only - http://bmjopen.bmj.com/site/about/guidelines.xhtml

demographic and co-morbidity variables, which were available from the CPRD dataset, allowed us to adjust for a number of potential confounding factors. However, as with most retrospective cohort studies, we were unable to study all known and unknown potential confounding variables and thus there could be residual confounding, which we could not control for.

Comparisons with other studies

A Dutch RCT among children aged 2-8 years who did not fulfil the Paradise criteria [4] for severe recurrent throat infection frequency, which found the effects of tonsillectomy were more pronounced in children who had 3-6 throat infections in the year prior to entering the trial than in those with ≤2 throat infections.[8] Among children in this latter group, the authors reported fewer throat infections (-0.21, 95% CI -0.06 to -0.36) and fewer sore throats (-0.60, 95% CI -0.30 to -0.90) per person year in the tonsillectomy group but concluded that these differences were not large enough to be clinically significant, although the median follow-up time was only 22 months. [8] Although ours was a cohort study, we similarly found that although tonsillectomy led to a statistically significant reduction in ATI consultation rates among children with low baseline ATI, this was not necessarily clinically significant.

A UK study which analysed CPRD data for annual sore throat consultation rates found that the rates per 1000 registered patients declined by 50% between 1995 and 2000 among children aged 5-16 years.[17] The authors reported sore throat consultation rates in 2000 of 59 and 76 per 1000 registered patients among children aged 5-10 and 11-16 years,

Field Code Changed

Field Code Changed

For peer review only - http://bmjopen.bmj.com/site/about/guidelines.xhtml

respectively. However, that study did not investigate the annual consultation rates for an individual child. The authors proposed that the overall decline in consultation rates may reflect that patients were increasingly self-managing minor illnesses. [17]

Field Code Changed

Field Code Changed

We found the natural history among children of all ages who did not have tonsillectomy was for a gradual increase in the number of ATI consultations. This is contrary to previous studies, which reported that the natural history of sore throats is to attenuate over time.[18] [19, 20] However, our study was based in a community setting and focused on a population of children with low baseline ATI who may have emerging natural history whereas other studies have selected children with higher baseline ATI from the outset. If we had followed children up for longer we may have observed a decline over time.

Clinical implications of the study

Overall, children aged 4-15 years, who underwent tonsillectomy compared with those who did not, experienced an 88% and 76% reduced risk of having ≥3 ATI consultations over 1-3 years and 4-6 years' follow-up, respectively. However, the observed decline in ATI consultations might also, in part, be attributed to adeno-tonsillectomy operations reducing rhino-sinusitis or otitis media infections, which were coded as URTIs.[21, 22] [23]

There was no evidence of an association between tonsillectomy and deprivation level, which is in contrast to some recent studies and reports.[24-27] However, we only had practice-level deprivation data and not individual-level data, which could account for the absence of an association.

For peer review only

Children in our study who underwent tonsillectomy in 2001 had ≤3 ATI consultations documented in general practice during the three years prior to their operation. This is markedly less than the minimum threshold recommended by national guidelines, reviews and the Paradise criteria for frequency of episodes prior to tonsillectomy. [1] [4] [20] [28] [29] These findings may reflect lower thresholds being followed or perhaps represent sub-optimal documentation of sore throat consultations prior to tonsillectomy in primary care settings. Although we assumed that parents would have consulted if the ATI was severe, the low consultation rate could reflect HSB, whereby some parents may not present with ATI episodes if, for example, antibiotics were not issued at prior consultations. However, this depends on the individual GP's attitudes and threshold for prescribing antibiotics for ATIs.

Other possible explanations for these low ATI consultation rates are that we did not have access to the 'free text' within individual medical consultation records, which may contain additional details about ATI episodes. Children may have attended with concurrent signs or symptoms and so other diagnostic codes, such as otitis media or specific viral URTIs, may have been recorded with details of the sore throat symptoms included within the 'free text'. As previously mentioned, overall annual consultation rates for sore throat declined by 50% among children aged 5-16 years between 1995 and 2000 in the UK, which may further account for our lower ATI consultation rates at baseline.[17] We did not have ~~or~~ consultations from other primary care settings, direct Accident and Emergency department attendances or hospital admissions, which have been increasing over recent years and ~~have been increasing in recent years~~may not be well-documented within general practice records.[30] ~~[18]~~Therefore, we interpret our findings with the caveat that there could be

Field Code Changed

Field Code Changed

For peer review only - http://bmjopen.bmj.com/site/about/guidelines.xhtml

other possible explanations for the low documentation rate of ATI consultations. However, the strength of our study is that we are consistent in comparing the ATI consultation frequency prior to and subsequent to the index date for each child in a particular general practice, which is where the vast majority of consultations with children occur.[10] ~~w~~ We recommend future studies should examine individual consultations in greater detail, as well as attendances in community and hospital emergency settings, to attempt to capture ATI consultations within different healthcare settings.

Our findings suggest that there is limited clinical benefit in performing tonsillectomy for children with low baseline ATI consultation rates. This reinforces existing guidance that only children with severe throat infection disease at baseline should be referred for tonsillectomy. Previous research has suggested that the operation is beneficial in the short-term for those with more serious ATI morbidity at baseline; although its benefit in the longer-term is still not clear and this still needs to be investigated. Tonsillectomy is a major operation so it is important that surgery confers significant individual and societal benefits, including fewer days off work by parents affected by sick children.

Our study also highlights the importance of diligent documentation of ATI consultations within general practice. There is a need to validate diagnostic screening tools among children, such as the Centor criteria and McIsaac scores, for group A β-haemolytic streptococcal throat infections. This may help towards more accurate diagnosis, recording and more appropriate management of ATI consultations in general practice.

For peer review only

For peer review only - http://bmjopen.bmj.com/site/about/guidelines.xhtml

~~Tonsillectomy is a major operation so it is important that surgery confers significant individual and societal benefits, including fewer days off work by parents affected by sick children. Previous research has suggested surgery is beneficial in the short-term for those with more serious ATI morbidity at baseline. However, its benefit in the longer-term is still not clear and this still needs to be investigated.~~

Conclusion

Our study found a statistically significant reduction in ATI consultation rates among children with low baseline ATI consultation rates who underwent tonsillectomy compared with those who did not. However, for this subgroup of children, the relatively modest reduction in ATI consultation rates in clinical terms may be outweighed by the potential risks related to the operation. The long-term clinical effectiveness of tonsillectomy for children, across the ATI frequency spectrum, still needs to be evaluated. This needs to be undertaken in conjunction with updated cost-effectiveness models, stratified by age, sex and baseline ATI rates, so that clinicians can risk profile children to determine which subgroups of children are most likely to benefit from surgery.

For peer review only - http://bmjopen.bmj.com/site/about/guidelines.xhtml

FUNDING: EK is funded by a National Institute for Health Research Doctoral Research Fellowship (2009-02-78). SS is funded by a a National Institute for Health Research Career Development Fellowship (NIHR CDF-2011-04-048). HW is funded by NIHR Research Design Service.This article presents independent research commissioned by the National Institute for Health Research (NIHR) under the Collaborations for Leadership in The views expressed in this publication are those of the author(s) and not necessarily those of the NHS, the NIHR or the Department of Health.

ETHICAL APPROVAL : The study was approved by the MHRA Independent Scientific Advisory Committee on Database Research

ACKNOWLEDGEMENTS: Data was obtained from the Clinical Practice Research Datalink, CPRD (formerly known as the General Practice Research Database). The CPRD is jointly funded by the NHS National Institute for Health Research (NIHR) and the Medicines and Healthcare products Regulatory Agency (MHRA). ~~EK is funded by a National Institute for Health Research Doctoral Research Fellowship. SS is funded by a National Institute for Health Research Career Development Fellowship. HW is funded by NIHR Research Design Service.~~

COMPETING INTERESTS: none declared

DATA SHARING STATEMENT: no additional data are available

CONTRIBUTOR STATEMENT: The study was conceived by EK, SS and MS. The study was designed by EK, SS, HW and AB. VC extracted the data. EK analysed the data. HW provided statistical advice. EK wrote the first draft of the manuscript. All authors contributed to the interpretation of the findings and revised the paper critically for intellectual content. All authors have seen and approved this version of the manuscript. EK and SS are the guarantors of this study.

For peer review only

REFERENCES

1. SIGN. Scottish Intercollegiate Guidelines Network 117. Management of sore throat and indications for tonsillectomy. . April 2010. http://www.sign.ac.uk/pdf/sign117.pdf.

2. Lock C, Wilson J, Steen N, et al. North of England and Scotland Study of Tonsillectomy and Adeno-tonsillectomy in Children(NESSTAC): a pragmatic randomised controlled trial with a parallel non-randomised preference study. Health Technol Assess. 2010 Mar;14(13):1-164, iii-iv.

3. Orvidas LJ, St Sauver JL, Weaver AL. Efficacy of tonsillectomy in treatment of recurrent group A beta-hemolytic streptococcal pharyngitis. Laryngoscope. 2006 Nov;116(11):1946-50.

4. Paradise JL, Bluestone CD, Bachman RZ, et al. Efficacy of tonsillectomy for recurrent throat infection in severely affected children. Results of parallel randomized and nonrandomized clinical trials. N Engl J Med. 1984 Mar 15;310(11):674-83.

5. Koshy E, Curcin V, Bottle A, et al. Sore throat consultations in general practice prior to tonsillectomy among eight hundred and sixty-three children in England: is this in accordance with the SIGN guidelines? Clin Otolaryngol. 2013 Jun;38(3):266-70.

6. Toh A, Mullin A, Grainger J, et al. Indications for tonsillectomy: are we documenting them? Annals of the Royal College of Surgeons of England. 2009 Nov;91(8):697-9.

For peer review only - http://bmjopen.bmj.com/site/about/guidelines.xhtml

7. Paradise JL, Bluestone CD, Colborn DK, et al. Tonsillectomy and adenotonsillectomy for recurrent throat infection in moderately affected children. Pediatrics. 2002 Jul;110(1 Pt 1):7-15.

8. van Staaij BK, van den Akker EH, Rovers MM, et al. Effectiveness of adenotonsillectomy in children with mild symptoms of throat infections or adenotonsillar hypertrophy: open, randomised controlled trial. BMJ. 2004 Sep 18;329(7467):651.

9. Tsou YA, Lin CC, Lai CH, et al. Does Adenotonsillectomy really reduced clinic visits for pediatric upper respiratory tract infections? A national database study in Taiwan. Int J Pediatr Otorhinolaryngol. 2013 May;77(5):677-81.

10. Hippisley-Cox J, Vinogradova Y. Trends in Consultation Rates in General Practice 1995/1996 to 2008/2009: Analysis of the QResearch® database. http://www.ic.nhs.uk/webfiles/publications/gp/Trends_in_Consultation_Rates_in_General_Practice_1995_96_to_2008_09.pdf. 2009.

11. Saxena S, Francis N, Sharland M. Primary care of children: the unique role of GPs. Br J Gen Pract. 2012 Jul;62(600):340-1.

12. CPRD. Clinical Practice Research Datalink. http://www.cprd.com/.

13. Parker NP, Walner DL. Trends in the indications for pediatric tonsillectomy or adenotonsillectomy. Int J Pediatr Otorhinolaryngol. 2011 Feb;75(2):282-5.

For peer review only

For peer review only - http://bmjopen.bmj.com/site/about/guidelines.xhtml

14. Ram G, Chinen J. Infections and immunodeficiency in Down syndrome. Clinical and experimental immunology. 2011 Apr;164(1):9-16.

15. Vestergaard H, Wohlfahrt J, Westergaard T, et al. Incidence of tonsillectomy in Denmark, 1980 to 2001. Pediatr Infect Dis J. 2007 Dec;26(12):1117-21.

16. Stata/SE version 11.1 http://www.stata.com/stata11/point1.html.

17. Ashworth M, Charlton J, Latinovic R, et al. Age-related changes in consultations and antibiotic prescribing for acute respiratory infections, 1995-2000. Data from the UK General Practice Research Database. Journal of clinical pharmacy and therapeutics. 2006 Oct;31(5):461-7.

18. Buskens E, van Staaij B, van den Akker J, et al. Adenotonsillectomy or watchful waiting in patients with mild to moderate symptoms of throat infections or adenotonsillar hypertrophy: a randomized comparison of costs and effects. Arch Otolaryngol Head Neck Surg. 2007 Nov;133(11):1083-8.

19. van Staaji BK, van den Akker EH, Rovers MM, et al. Effectiveness of adenotonsillectomy in children with mild symptoms of throat infections or adenotonsillar hypertrophy: open, randomised controlled trial. Clin Otolaryngol. 2005 Feb;30(1):60-3.

20. Burton MJ, Glasziou PP. Tonsillectomy or adeno-tonsillectomy versus non-surgical treatment for chronic/recurrent acute tonsillitis. Cochrane Database Syst Rev. 2009(1):CD001802.

For peer review only

For peer review only - http://bmjopen.bmj.com/site/about/guidelines.xhtml

21. van den Aardweg MT, Schilder AG, Herkert E, et al. Adenoidectomy for otitis media in children. Cochrane Database Syst Rev. 2010(1):CD007810.

22. Paradise JL, Bluestone CD, Colborn DK, et al. Adenoidectomy and adenotonsillectomy for recurrent acute otitis media: parallel randomized clinical trials in children not previously treated with tympanostomy tubes. JAMA. 1999 Sep 8;282(10):945-53.

23. Darrow DH, Siemens C. Indications for tonsillectomy and adenoidectomy. Laryngoscope. 2002 Aug;112(8 Pt 2 Suppl 100):6-10.

24. Bisset AF, Russell D. Grommets, tonsillectomies, and deprivation in Scotland. BMJ. 1994 Apr 30;308(6937):1129-32.

25. Croxford R, Friedberg J, Coyte PC. Socio-economic status and surgery in children: myringotomies and tonsillectomies in Ontario, Canada, 1996-2000. Acta paediatrica (Oslo, Norway : 1992). 2004 Sep;93(9):1245-50.

26. Koshy E, Bottle A, Murray J, et al. Changing indications and socio-demographic determinants of (adeno)tonsillectomy among children in England--are they linked? A retrospective analysis of hospital data. PLoS One. 2014;9(8):e103600.

27. Chief Medical Officer's Annual Report (2005) Department of Health. http://www.dh.gov.uk/prod_consum_dh/groups/dh_digitalassets/@dh/@en/documents/digitalasset/dh_4137367.pdf.

For peer review only

28. Baugh RF, Archer SM, Mitchell RB, et al. Clinical practice guideline: tonsillectomy in children. Otolaryngol Head Neck Surg. 2011 Jan;144(1 Suppl):S1-30.

29. SIGN. Scottish Intercollegiate Guidelines Network 34: – Management of sore throat and indications for tonsillectomy. Available on specific request from SIGN, as it is no longer directly accessible from the SIGN website. (1999) http://www.atb.healthnet.sk/Sore_Throat/sign34-sore-throat.pdf (accessed 15th October 2014).

30. Koshy E, Murray J, Bottle A, et al. Significantly increasing hospital admissions for acute throat infections among children in England: is this related to tonsillectomy rates? Arch Dis Child. 2012 Dec;97(12):1064-8.

For peer review only

S1: Flow diagram of cohort construction

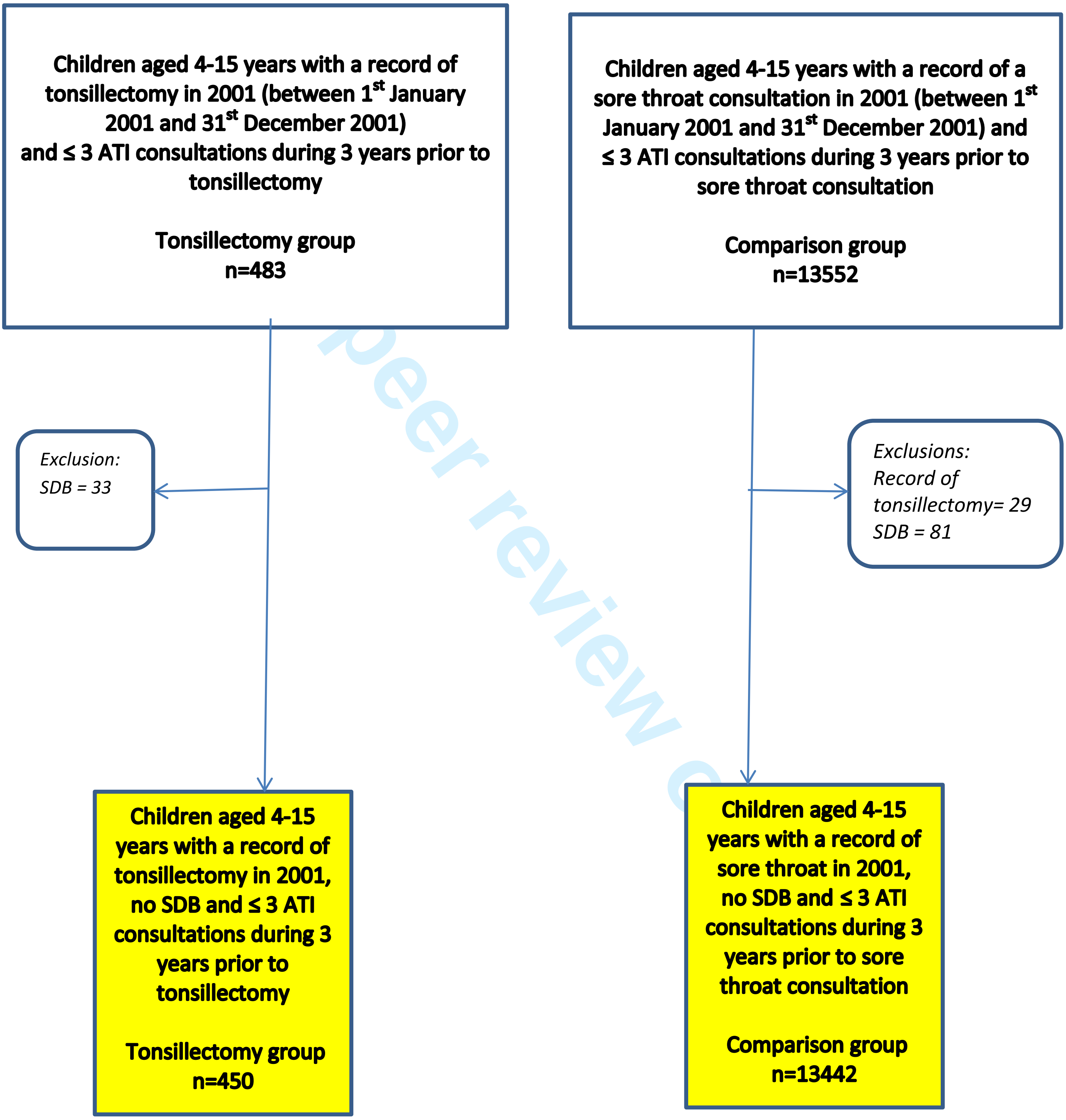

ATI- acute throat infection; SDB – sleep- disordered breathing

For peer review only - http://bmjopen.bmj.com/site/about/guidelines.xhtml

S2 – Read codes

Sore throat

Acute tonsillitis	H03
Acute erythematous tonsillitis	H030.
Acute follicular tonsillitis	H031.
Acute ulcerative tonsillitis	H032.
Acute catarrhal tonsillitis	H033.
Acute gangrenous tonsillitis	H034.
Acute bacterial tonsillitis	H035.
Acute viral tonsillitis	H036.
Acute tonsillitis NOS	H03z
streptococcal tonsillitis	A3403
Acute staphylococcal tonsillitis	H0351
Acute pneumococcal tonsillitis	H0350
O/E - exudate on tonsils	2DB7.

Acute pharyngitis	H02.
Acute gangrenous pharyngitis	H020.
Acute ulcerative pharyngitis	H022.
Acute bacterial pharyngitis	H023.
Acute viral pharyngitis	H024.
Acute staphylococcal pharyngitis	H0231
Acute pharyngitis NOS	H02z
Acute bacterial pharyngitis NOS	H023z
Streptococcal pharyngitis	A3402
Acute pneumococcal pharyngitis	H0230
Acute phlegmonous pharyngitis	H021.
Acute nasopharyngitis	H00

For peer review only

For peer review only - http://bmjopen.bmj.com/site/about/guidelines.xhtml

Streptococcal sore throat	A340.
Has a sore throat	1C92.
Persistent sore throat	1C93.
Sore throat symptom NOS	1C9Z.
Streptococcal sore throat NOS	A340z
throat pain	1CB3.
[D]Throat pain	R041.
Throat soreness	1C9..
Has a sore throat	1C92.

Upper respiratory tract infections

H1z..00	Upper respiratory tract disease NOS
H051.00	Acute upper respiratory tract infection
H05z.11	Upper respiratory tract infection NOS
H05..00	Other acute upper respiratory infections
H05z.12	Viral upper respiratory tract infection NOS
H05z.00	Upper respiratory infection NOS

For peer review only - http://bmjopen.bmj.com/site/about/guidelines.xhtml

STROBE 2007 (v4) checklist of items to be included in reports of observational studies in epidemiology*
Checklist for cohort, case-control, and cross-sectional studies (combined)

Section/Topic	Item #	Recommendation	Reported on page #
**Title and abstract**	1	(*a*) Indicate the study's design with a commonly used term in the title or the abstract	1
		(*b*) Provide in the abstract an informative and balanced summary of what was done and what was found	2-3
**Introduction**			
Background/rationale	2	Explain the scientific background and rationale for the investigation being reported	5
Objectives	3	State specific objectives, including any pre-specified hypotheses	5-6
**Methods**			
Study design	4	Present key elements of study design early in the paper	6-7
Setting	5	Describe the setting, locations, and relevant dates, including periods of recruitment, exposure, follow-up, and data collection	6-8
Participants	6	(*a*) *Cohort study*—Give the eligibility criteria, and the sources and methods of selection of participants. Describe methods of follow-up<br>*Case-control study*—Give the eligibility criteria, and the sources and methods of case ascertainment and control selection. Give the rationale for the choice of cases and controls<br>*Cross-sectional study*—Give the eligibility criteria, and the sources and methods of selection of participants	6-7
		(*b*) *Cohort study*—For matched studies, give matching criteria and number of exposed and unexposed<br>*Case-control study*—For matched studies, give matching criteria and the number of controls per case	n/a
Variables	7	Clearly define all outcomes, exposures, predictors, potential confounders, and effect modifiers. Give diagnostic criteria, if applicable	8-9
Data sources/ measurement	8*	For each variable of interest, give sources of data and details of methods of assessment (measurement). Describe comparability of assessment methods if there is more than one group	*8-9*
Bias	9	Describe any efforts to address potential sources of bias	7-9, 15
Study size	10	Explain how the study size was arrived at	6
Quantitative variables	11	Explain how quantitative variables were handled in the analyses. If applicable, describe which groupings were chosen and why	8-9
Statistical methods	12	(*a*) Describe all statistical methods, including those used to control for confounding	8-9
		(*b*) Describe any methods used to examine subgroups and interactions	8-9
		(*c*) Explain how missing data were addressed	9
		(*d*) *Cohort study*—If applicable, explain how loss to follow-up was addressed<br>*Case-control study*—If applicable, explain how matching of cases and controls was addressed	Discussed p15

For peer review only

		Cross-sectional study—If applicable, describe analytical methods taking account of sampling strategy	
		(*e*) Describe any sensitivity analyses	
**Results**			
Participants	13*	(a) Report numbers of individuals at each stage of study—eg numbers potentially eligible, examined for eligibility, confirmed eligible, included in the study, completing follow-up, and analysed	6-7, 10, Tables 1 and 2 and Supplementary figure S1
		(b) Give reasons for non-participation at each stage	10 and Supplementary figure S1
		(c) Consider use of a flow diagram	Supplementary figure S1
Descriptive data	14*	(a) Give characteristics of study participants (eg demographic, clinical, social) and information on exposures and potential confounders	11 (Table 1)
		(b) Indicate number of participants with missing data for each variable of interest	9,11
		(c) *Cohort study*—Summarise follow-up time (eg, average and total amount)	10
Outcome data	15*	*Cohort study*—Report numbers of outcome events or summary measures over time	12-14
		Case-control study—Report numbers in each exposure category, or summary measures of exposure	
		Cross-sectional study—Report numbers of outcome events or summary measures	
Main results	16	(*a*) Give unadjusted estimates and, if applicable, confounder-adjusted estimates and their precision (eg, 95% confidence interval). Make clear which confounders were adjusted for and why they were included	12-14
		(*b*) Report category boundaries when continuous variables were categorized	11
		(*c*) If relevant, consider translating estimates of relative risk into absolute risk for a meaningful time period	12-14
Other analyses	17	Report other analyses done—eg analyses of subgroups and interactions, and sensitivity analyses	
**Discussion**			
Key results	18	Summarise key results with reference to study objectives	14
Limitations	19	Discuss limitations of the study, taking into account sources of potential bias or imprecision. Discuss both direction and magnitude of any potential bias	15
Interpretation	20	Give a cautious overall interpretation of results considering objectives, limitations, multiplicity of analyses, results from similar studies, and other relevant evidence	16-19
Generalisability	21	Discuss the generalisability (external validity) of the study results	16-17
**Other information**			
Funding	22	Give the source of funding and the role of the funders for the present study and, if applicable, for the original study on which the present article is based	21

For peer review only

*Give information separately for cases and controls in case-control studies and, if applicable, for exposed and unexposed groups in cohort and cross-sectional studies.

Note: An Explanation and Elaboration article discusses each checklist item and gives methodological background and published examples of transparent reporting. The STROBE checklist is best used in conjunction with this article (freely available on the Web sites of PLoS Medicine at http://www.plosmedicine.org/, Annals of Internal Medicine at http://www.annals.org/, and Epidemiology at http://www.epidem.com/). Information on the STROBE Initiative is available at www.strobe-statement.org.

For peer review only
